# Transcatheter versus surgical aortic valve replacement in low-risk patients with severe aortic stenosis: a systematic review and meta-analysis

**DOI:** 10.1186/s12872-026-05558-6

**Published:** 2026-02-19

**Authors:** Ziad A. Fadl, Moaz Yasser Darwish, Walaa M. Moawad, Nada Osama Aboelmagd, Ganna Abdelrhman Fadl, Mostafa Wanees Ahmed El Husseny, Nada K. Abdelsattar, Taha Abd-ElSalam Ashraf Taha

**Affiliations:** 1 Faculty of Medicine, Fayoum University, Fayoum, Egypt; 2 Faculty of Medicine, Qena University, Qena, Egypt; 3 Department of Adult Cardiology, Aswan Heart Centre, Aswan, Egypt; 4 Department of Cardiology, Faculty of medicine, Fayoum university, Egypt

**Keywords:** Aortic stenosis, Low risk, TAVR, SAVR

## Abstract

**Background:**

The optimal revascularization strategy for low-risk patients with severe aortic stenosis (AS), transcatheter (TAVI) versus surgical aortic valve replacement (SAVR), remains a subject of ongoing debate, particularly regarding long-term outcomes. We aimed to synthesize the evidence on the comparative safety and efficacy of TAVI and SAVR in this population.

**Methods:**

We conducted a systematic review and meta-analysis, searching PubMed, Scopus, Web of Science, and Cochrane Library until March 2025. We included randomized controlled trials (RCTs) and comparative observational studies enrolling low-risk patients with severe AS. Primary outcomes were all-cause mortality and stroke, analyzed at different time points. Data were pooled using random-effects models, with subgroup analyses based on study design.

**Results:**

Thirty studies, including 48,210 patients, were included. TAVI was associated with a significantly lower risk of 30-day mortality (Relative Risk [RR] 0.66, 95% CI 0.50–0.76) and 30-day stroke (RR 0.70, 95% CI 0.53–0.92). However, in the overall analysis, SAVR was favored for one-year mortality (RR 1.11, 95% CI 1.01–1.21), with equipoise in long-term follow-up. TAVI significantly reduced the risks of 30-day major bleeding (RR 0.38, 95% CI 0.21–0.70) and atrial fibrillation (RR 0.41, 95% CI 0.22–0.76). Conversely, TAVI was associated with a persistently higher risk of permanent pacemaker implantation (RR 2.44 at 30 days, 95% CI 1.76–3.40) and paravalvular leakage (RR > 5-fold at both 30 days and 1 year).

**Conclusion:**

In low-risk patients with severe AS, TAVI offers clear early procedural safety advantages, particularly regarding stroke, bleeding, and atrial fibrillation. While the overall analysis suggested a 30-day mortality benefit for TAVI and a 1-year mortality benefit for SAVR, these findings were driven principally by observational data and were not statistically significant in the subgroup of randomized controlled trials. These procedural benefits are counterbalanced by a persistently higher risk of permanent pacemaker implantation and paravalvular leakage with TAVI. The choice of intervention requires a nuanced, individualized approach through shared decision-making, weighing early recovery benefits against device-related risks and the divergence between trial and registry outcomes.

**Supplementary Information:**

The online version contains supplementary material available at 10.1186/s12872-026-05558-6.

## Introduction

Aortic stenosis (AS) is a progressive, degenerative disease that ranges from asymptomatic leaflet calcification to severe obstruction that ultimately leads to pump failure [1]. It is particularly common among older population, with a prevalence of about 4% in those above the age of 85. In the natural history for AS, almost all affected individuals ultimately need valve replacement [2]. Surgical aortic valve replacement (SAVR) has long been the standard therapy for severe aortic stenosis [3]. The development of Transcatheter aortic valve implantation (TAVI) as a less invasive alternative to open-heart surgery represented a paradigm shift in the management of AS with an emerging expansion of its indication over recent years.

The choice between SAVR and TAVI is a complex procedure that requires a mandatory multidisciplinary heart team discussion that include patient risk profile assessment as well as clinical and anatomical criteria for TAVI eligibility. European guidelines have expanded indications for TAVI, recommending it for patients older than 75 years regardless of surgical risk and as an alternative to SAVR in those at high or intermediate risk [4].

Low surgical risk is typically defined by a Society of Thoracic Surgeons (STS) score below 4%. Several recent trials have reported favorable early outcomes for low-risk patients undergoing TAVI. The PARTNER 3 trial demonstrated that TAVI is non-inferior to SAVR for mortality and stroke at two years [5], and the Evolut low-risk study showed comparable lengths of stay between the two procedures [6]. Conversely, a 2023 meta-analysis suggested higher rates of all-cause mortality and aortic regurgitation with TAVI [7]. Despite these data, TAVI has not yet been fully integrated into clinical decision-making for low-risk patients with symptomatic severe aortic stenosis [8].

Given the ongoing debate about whether TAVI offers greater benefit than SAVR in low-risk patients over extended follow-up periods, we performed an updated systematic review and meta-analysis. Our aim was to compare postoperative outcomes between TAVI and SAVR, in low-risk patients focusing on clinical safety and long-term efficacy.

## Methods

We performed this systematic review and meta-analysis following the guidelines outlined in the preferred reporting Items for systematic review and Meta-analysis (PRISMA) statement [9, 10].

### Eligibility criteria

Studies were regarded as eligible if they include: (1) Adult patients 18 years or older with severe aortic stenosis and low surgical risk. Patients are considered low surgical risk if they are less than 4% in the Society of Thoracic Surgeons Score “STS score”, Or less than 5% according to the Logistic Euro Score I of less than 10% according to Logistic Euro Score II. (2) Focused on Trans-catheter aortic valve replacement or implantation (3) Compared to Surgical aortic valve replacement. (4) Studies must be randomized controlled trials or comparative observational cohort studies.

Studies were excluded if they are: (1) Animal studies, (2) Non-English publications, (3) Case reports or case series, (4) Single-arm studies, (5) Review articles, (6) Conference abstracts, (7) In-vitro studies.

A comprehensive search was done across four databases: PubMed, Scopus, Web of Science and Cochrane Library from database inception till March 2025. The following Search strategy was used: (“transcatheter aortic valve replacement” OR “transcatheter aortic valve implantation” OR TAVR OR TAVI) AND (“surgical aortic valve replacement” OR “surgical aortic valve implantation” OR SAVR OR SAVI) AND (“low-risk” OR “low risk” OR “low surgical risk”).

The retrieved studies were initially handled using EndnoteX9 reference management software and then exported to Microsoft Excel for screening. Two stages of Screening were performed; (1) Initial assessment of titles and abstracts to identify relevant studies. (2) comprehensive full text reviewing of the selected articles to ensure their eligibility. Screening was done by two independently reviewers, and conflicts between them was resolved by consultation of a third one.

## Date extraction

Data extraction was performed by two independent authors using a standardized pre-piloted form. The data extracted included: (1) General study characteristics: authors, publication year, country, study design, and sample size; (2) Demographics and comorbidities: age, sex, diabetes mellitus, hypertension, prior myocardial infarction, and lung disease; (3) Baseline characteristics: surgical risk scores (STS or EuroSCORE), baseline aortic valve area, and ejection fraction; (4) Procedural details: valve type, generation, and access route; and (5) Outcomes: all-cause mortality, stroke, major bleeding, acute kidney injury (AKI), new-onset atrial fibrillation, permanent pacemaker implantation (PPI), paravalvular leakage (PVL), valve thrombosis, aortic reintervention, hospital length of stay, and hemodynamic parameters (mean gradient, aortic valve area).

To ensure consistency, outcome events such as PPI and bleeding were extracted according to the definitions provided in the primary studies (predominantly VARC-2). Furthermore, hemodynamic data were strictly categorized as either ‘post-procedural’ or ‘change-from-baseline’ to prevent data mixing.

## Quality assessment

Quality assessment of included studies was performed independently by two reviewers. The Newcastle-Ottawa Scale (NOS) was employed to evaluate the risk of bias in cohort studies and non-randomized clinical trials [11]. The NOS uses a star-rating system to assess selection, comparability, and outcome assessment. For randomized controlled trials, the Risk of Bias 2 (ROB2) tool was applied [12]. The ROB2 assesses risk of bias across five domains: bias arising from the randomization process, bias due to deviations from intended interventions, bias due to missing outcome data, bias in measurement of the outcome, and bias in selection of the reported result. Any disagreements between reviewers were resolved through discussion and, if necessary, consultation with a senior reviewer. The Grading of Recommendations Assessment, Development and Evaluation (GRADE) approach was applied to evaluate the overall certainty of the evidence for each outcome generated by our meta-analysis, categorizing the certainty as high, moderate, low, or very low [13].

## Statistical analysis and data synthesis

The extracted data were analyzed using RevMan software (version 5.4) for Windows. Post-intervention and change from baseline in ejection fraction, aortic valve area and aortic valve pressure gradients for was calculated whenever feasible and pooled in meta-analysis models as mean difference using the Inverse Variance method. The rest of outcomes were presented as dichotomous data which were pooled as relative risk using the Mantel–Haenszel (M–H) method.

Fixed effect model was adopted whenever pooled studies were homogamous, however, random effect model was the adopted one whenever pooled studies were observed to be heterogenous. A significant result was concluded whenever the *P* value was less than 0.05 [14].

In dichotomous outcomes, studies that reported no events in either of the two arms were deemed to be unsuitable for meta-analysis. Visual inspection of the forest plot was the primary method of identifying heterogeneity between pooled studies in a meta-analysis model. Whenever suspected, heterogeneity was measured using Chi-Square and I-Square tests and confirmed by a Chi-square P value of less than 0.05. Funnel plots were generated for the primary outcomes (mortality and stroke) whenever the meta-analysis model included ten studies or more to explore the potential presence of small-study effects and publication bias through visual inspection.

To avoid double-counting, when multiple reports from the same trial existed, we selected the report corresponding exactly to the time point of interest. For the overall long-term analysis, we used the longest available follow-up for each unique cohort.

## Results

**Fig. 1:**
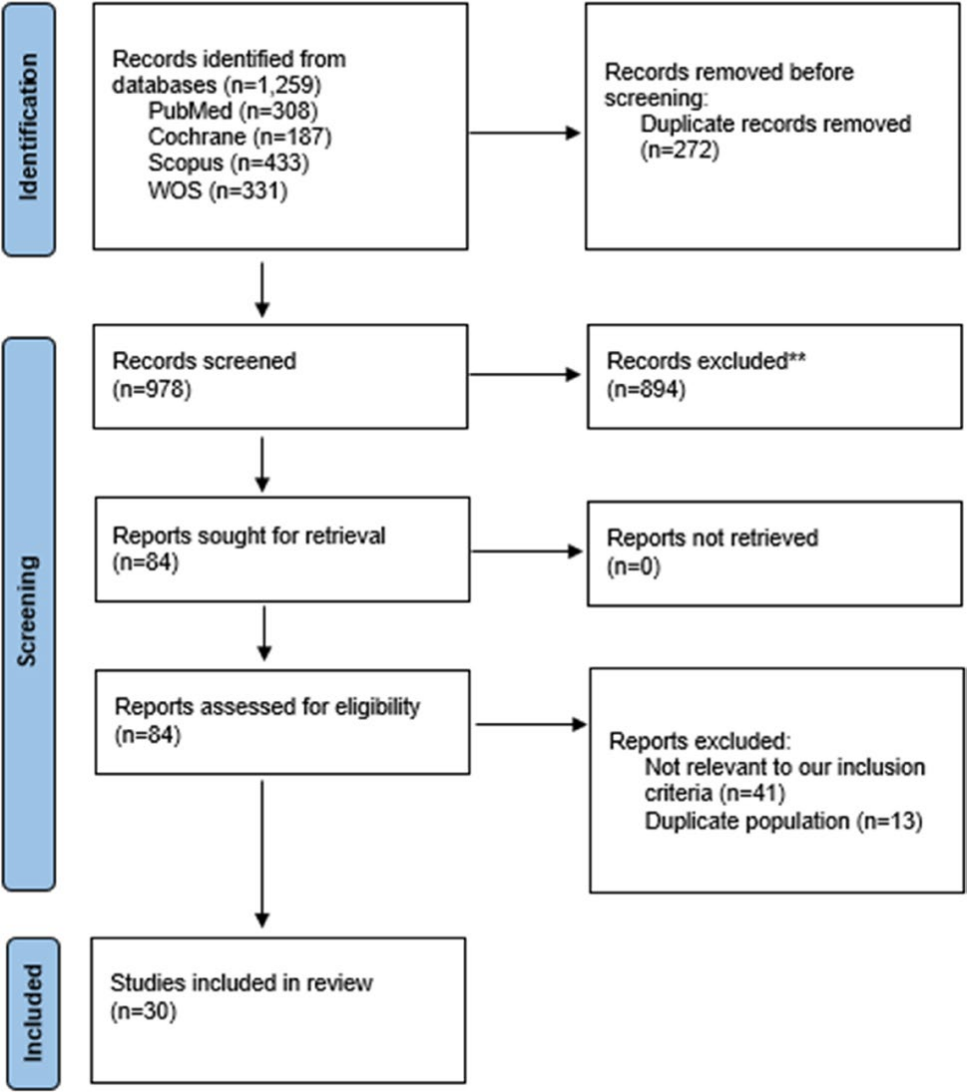
PRISMA flow chart of the included studies

Our systematic search retrieved 1,259 records from PubMed, Cochrane, Scopus and Web od science (WOS). EndNote X9 identified and removed 272 duplicate records before title and abstract screening. Full-text screening included 84 records which their titles and abstracts were found to be possibly relevant to our inclusion criteria. Finally, 30 [15–44] studies were included in the current systematic review and meta-analysis. Study selection process is illustrated within Fig. 1.

### General and baseline characteristics of the included studies

The current research work included 18 cohort studies [15–32], 11 randomized controlled trials [34–44] and 1 case-control study [33] comparing TAVI with SAVR in low-risk patients with severe aortic stenosis. Four reports of NOTION (Nordic Aortic Valve Intervention) trial [34, 37–39], 2 reports of PARTNER 3 trial [35, 44] and 2 reports of Evolut rrial [36, 43] were included, in addition to 2 reports from the trials of Virtanen et al. [20, 21]. The TAVI sample sizes ranged from 20 to 7,605 participants, with most studies enrolling between 140 and 700 patients, and each included a contemporary SAVR comparator. Mean age of the TAVI groups varied from 69 to 84 years, and men were approximately half to two-thirds of participants across most studies. Baseline surgical risk scores were low, with mean STS or EuroSCORE values typically between 1.9% and 3% respectively, although a few early cohorts reported higher EuroSCOREs (~ 8–9%). The devices used included both of balloon-expandable valves (SAPIEN XT or SAPIEN 3) and self-expanding valves (CoreValve/Evolut R/PRO). Follow-up durations spanned 6–120 months, although most studies reported outcomes at 12–60 months. Table 1 summarizes general characteristics of the included studies.

**Table 1:** Summary of the general characteristics of the included studies

Study (year)	Design	Sites	TAVI (*n*)	SAVR (*n*)	Mean age (years)	Male *n* (%)	Mean STS/Euro score	TAVR device type (short)	Follow-up (months)
Mack et al. 2023 [44]	RCT	United States, New Zealand, Japan, Australia & Canada	496	454	73.3	335 (67.5)	1.9	Bioprosthetic valve (SAPIEN 3)	60
Mack et al. 2019 [35]	RCT	United States, New Zealand, Japan, Australia & Canada	496	454	73.3	335 (67.5)	1.9	Bioprosthetic valve (SAPIEN 3)	12
Popma et al. 2019 [36]	RCT	Australia, Canada, France, Japan, Netherlands, New Zealand & USA	734	734	74.0	468 (63.8)	1.9	Self-expanding supraannular bioprosthesis	24
Jørgensen et al. 2024 [34]	RCT	Denmark, Norway, Sweden, Finland & Iceland	187	183	71.1	119 (63.6)	1.15	Self-/balloon-expandable transcatheter valves	24
Virtanen et al. 2019 (matched) [20]	Retrospective study	Finland	304	304	77.9	143 (47.0)	2.1	Bioprosthesis	36
Handa et al. 2024 [27]	Retrospective multicenter registry	Japan	159	159	79.0	73 (47.8)	2.56	Balloon-/self-expanding	37
Biancari et al. 2024 [24]	Prospective cohort	Italy	355	355	80.1	206 (58.0)	2.55	CoreValve & Sapien XT	120
Mehaffey et al. 2024 [16]	Retrospective study	United States	7605	8144	69.7	4676 (61.5)	–	–	36
Blackmanet al. 2024 [42]	Multicenter international randomized	UK	730	684	74.0	475 (65.0)	–	Self-expanding (CoreValve/Evolut)	48
Lin et al. 2023 [25]	Retrospective cohort	Taiwan	20	37	79.6	9 (45.0)	2.6	CoreValve & Sapien	TAVI 40.8 ± 27.6/SAVR 56.4 ± 38.4
Bavry et al. 2023 [41]	Randomized parallel	USA	503	497	–	–	–	–	12
Ito et al. 2023 [40]	Post-hoc (Evolut low-risk trial)	USA	730	684	74.1	409 (61.6)	1.9	Self-expanding (CoreValve/Evolut)	24
Kolar et al. 2022 [28]	Retrospective single-center observational	Slovenia	53	53	–	–	2.5	Sapien XT/3, Evolut R, Portico	56
Maeda et al. 2022 [15]	Retrospective study	Japan	86	383	77.6	33 (38.0)	2.9	Self-/balloon-expandable valve	60
Saito et al. 2022 [17]	Retrospective study	Japan	66	121	–	–	3.0	–	24
Zubarevich et al. 2022 [22]	Retrospective study	USA & Italy	56	56	74.3	42 (75.0)	3.7	SAPIEN XT/3	6
Vilalta et al. 2021 [19]	Prospective study	Spain	171	171	77.4	62 (36.3)	2.6	Perceval/Sapien 3/Evolut PRO/Portico/Acurate neo	24
Brizido et al. 2021 [26]	Retrospective non-randomized cohort	Portugal	199	544	81.0	29 (49.0)	2.29	Evolut/Portico/Acurate neo/Sapien/Lotus	54
Blanke et al. 2020 [30]	Substudy (Evolut low-risk trial)	USA, Canada, Japan	197	178	74.0	128 (65.0)	–	Self-expanding (CoreValve/Evolut)	12
Bekeredjian et al. 2019 [23]	Prospective cohort	Germany	6062	14,487	78.87	3689 (60.9)	2.11	–	12
Schaefer et al. 2019 (matched) [18]	Retrospective study	Germany	109	109	–	–	2.0	Self-/balloon-expandable	60
Kowalowka et al. 2024 [31]	Multicentre registry analysis	Poland	329	593	–	–	2.46	Evolut R 56.5%, Sapien 3 18.8%, Symetis 17.3%	32.6
Muneretto et al. 2023 (matched) [33]	Case-control study	USA, Italy & Switzerland	346	346	80.0	157 (45.4)	2.5	Bioprostheses	60
Chung et al. 2023 [29]	Post-hoc	USA, Canada, France, Netherlands, Japan, Australia & New Zealand	332	287	74.7	217 (65.4)	1.95	Self-expanding bioprosthesis	24
Bo et al. 2019 [32]	Prospective cohort	Italy	32	20	79.8	15 (46.9)	3.03	–	12
Ramlawi et al. 2024 [43]	Post-hoc (prospective multinational randomized)	USA, Canada, Australia, Japan & several European countries	729	563	74.1	463 (63.5)	1.95	Self-expanding (CoreValve/Evolut)	48
Thyregod et al. 2015 [37]	RCT	Denmark & Sweden	145	135	79.2	78 (53.8)	2.9	CoreValve or surgical bioprosthesis	12
Thyregod et al. 2019 [38]	RCT	Denmark & Sweden	145	135	79.2	78 (53.8)	2.9	CoreValve or surgical bioprosthesis	60
Thyregod et al. 2024 [39]	RCT	Denmark & Sweden	145	135	79.2	78 (53.8)	2.9	CoreValve or surgical bioprosthesis	120
Virtanen et al. 2020 (matched) [21]	Retrospective study	Finland	140	140	76.5	61 (43.6)	2.0	Balloon/self-/mechanical; Perimount/Trifecta	36

### Comorbidities and hemodynamic profile of the included population

Comorbidities reflected a low-risk population yet showed some heterogeneity: diabetes mellitus was present in roughly 4–35% of participants, prior myocardial infarction in 5–27%, and chronic lung disease in 5–49%. History of atrial fibrillation was reported in a minority of studies, yet ranging widely from < 1% to about 15%. NYHA class III/IV symptoms were present in about 25% of patients, with one small series reporting a higher prevalence. A creatinine level > 2 mg/dL was rarely reported. Baseline aortic valve areas were consistently ≤ 0.8 cm² and mean gradients ranged from 44 to 55 mm Hg, confirming severe stenosis. Table 2 summarizes comorbidities and hemodynamic profile of the included population.

**Table 2:** Summary of summarizes comorbidities and hemodynamic profile of the included population

Study (year)	Prior MI (%)	Prior stroke (%)	Chronic lung disease (%)	Diabetes mellitus (%)	Creatinine > 2 mg/dL (%)	NYHA III/IV (%)	AF (%)	AVA (cm²)	Mean gradient (mmHg)
Mack et al. 2023 [44]	5.7	3.4	5.1	31.2	0.2	31.2	15.7	0.8	49.4
Mack et al. 2019 [35]	5.7	3.4	5.1	31.2	0.2	31.2	15.7	0.8	49.4
Popma et al. 2019 [36]	15.1	84.9	7.6	31.1	–	15.5	10.1	0.8	47.2
Jørgensen et al. 2024 [34]	7.0	–	1.1	19.8	–	17.1	68.4	0.7	50.4
Virtanen et al. 2019 (matched) [20]	18.8	35.2	1.0	5.1	–	12.8	1.6	–	–
Handa et al. 2024 [27]	6.9	–	5.0	4.4	–	13.2	71.7	68.3	48.7
Biancari et al. 2024 [24]	7.3	–	10.1	14.9	–	–	–	–	27.1
Mehaffey et al. 2024 [16]	13.1	–	8.4	22.9	–	8.7	5.7	–	–
Blackman et al. 2024 [42]	–	–	–	–	–	–	–	–	–
Lin et al. 2023 [25]	10.0	–	0.0	20.0	–	20.0	70.0	59.3	54.4
Bavry et al. 2023 [41]	–	–	–	–	–	–	–	–	–
Ito et al. 2023 [40]	14.7	84.8	7.6	29.8	–	15.1	9.9	64.5	0.8
Kolar et al. 2022 [28]	–	–	–	–	–	–	–	–	–
Maeda et al. 2022 [15]	9.0	42.0	19.0	21.0	–	5.0	17.0	66.0	0.68
Saito et al. 2022 [17]	–	–	–	–	–	–	–	–	–
Zubarevich et al. 2022 [22]	26.8	30.4	26.8	35.7	–	37.5	92.9	28.8	51.9
Vilalta et al. 2021 [19]	8.2	5.3	12.9	34.5	–	8.2	25.2	0.71	48.4
Brizido et al. 2021 [26]	18.0	–	48.0	32.0	–	11.0	44.0	–	26.7
Blanke et al. 2020 [30]	6.1	0.5	22.8	31.0	–	9.6	85.3	–	–
Bekeredjian et al. 2019 [23]	10.5	–	18.8	21.4	–	86.9	–	0.74	44.06
Schaefer et al. 2019 (matched) [18]	–	–	–	–	–	–	–	–	–
Kowalowka et al. 2024 [31]	–	–	–	–	–	–	–	–	–
Muneretto et al. 2023 (matched) [33]	9.0	–	23.7	24.0	–	4.6	84.1	1.81	56.4
Chung et al. 2023 [29]	3.0	–	16.6	29.8	–	–	–	–	30.8
Bo et al. 2019 [32]	–	–	–	–	–	–	–	51.8	50.9
Ramlawi et al. 2024 [43]	15.2	84.8	7.5	31.3	–	15.4	10.2	–	61.7
Thyregod et al. 2015 [37]	11.7	71.0	4.1	17.9	–	27.8	16.6	–	–
Thyregod et al. 2019 [38]	11.7	71.0	4.1	17.9	–	27.8	16.6	–	–
Thyregod et al. 2024 [39]	11.7	71.0	4.1	17.9	–	27.8	16.6	–	–
Virtanen et al. 2020 (matched) [21]	7.9	–	0.7	25.0	–	–	–	–	29.0

### Quality assessment

**Fig. 2:**
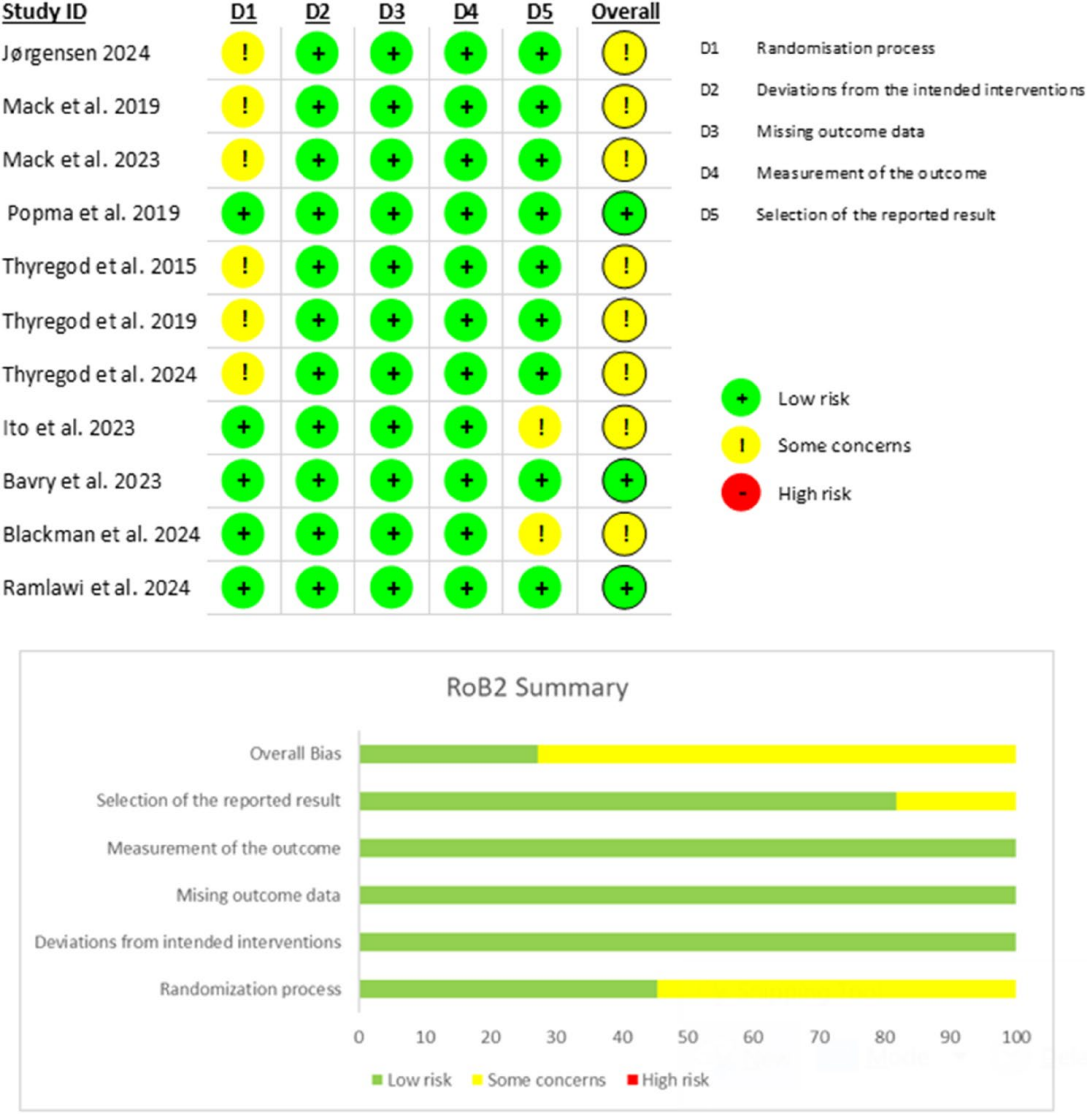
Risk of Bias 2 (RoB2) graph and summary of the included randomized controlled trials

The risk of bias in the included studies was assessed using appropriate tools based on study design. For randomized controlled trials (RCTs; *n* = 11), the Risk of Bias 2 (ROB2) tool was employed. Of the 11 RCTs, 3 were judged to be at low risk of bias [36, 41, 43], and 8 to have some concerns regarding bias [34, 35, 37–40, 42, 44]. The specific domains contributing to these judgments are detailed in Fig. 2. Cohort studies (*n* = 18) and case-control studies (*n* = 1) were evaluated using the Newcastle-Ottawa Scale (NOS). Based on the NOS assessment, two cohort studies were classified as poor quality [17, 28] and two as fair quality [18, 27] while the rest of studies were judged as good quality. The NOS scores for cohort and case-control studies are presented in supplementary Tables 1 and 2, respectively. The quality of our evidence-based results ranged from High to Very low with the detailed GRADE assessment being illustrated in supplementary Table 3.

### Outcomes

The pooled results for all clinical and hemodynamic outcomes are summarized in Table 3.

**Table 3:** Summary of Meta-Analysis findings for TAVI versus SAVR

Outcome	No. of Studies (k)	Effect Estimate (95% CI)	*P*-value (Effect)	Heterogeneity (I², *P*-value)
Mortality				
30-day	16	RR: 0.66 (0.50–0.76)	< 0.001	35%, *P* = 0.08
1-year	12	RR: 1.11 (1.01–1.21)	0.03	20%, *P* = 0.25
Long-term (2–10 years)	13	RR: 1.10 (0.89–1.36)	0.36	78%, *P* < 0.001
Stroke				
30-day	13	RR: 0.70 (0.53–0.92)	0.01	0%, *P* = 0.52
1-year	7	RR: 0.94 (0.71–1.26)	0.69	43%, *P* = 0.11
Long-term (2–5 years)	7	RR: 0.88 (0.72–1.07)	0.20	31%, *P* = 0.19
Permanent Pacemaker Implantation				
30-day	13	RR: 2.44 (1.76–3.40)	< 0.001	75%, *P* < 0.001
1-year*	7	RR: 2.33 (1.80–3.02)	< 0.001	34%, *P* = 0.17
Valve Thrombosis (1-year)	4	RR: 2.12 (0.73–6.12)	0.17	0%, *P* = 0.49
Aortic Reintervention				
30-day	4	RR: 0.35 (0.13–0.92)	0.03	0%, *P* = 0.45
1-year	4	RR: 1.04 (0.49–2.20)	0.93	0%, *P* = 0.80
Paravalvular Leakage				
30-day*	3	RR: 7.56 (6.04–9.47)	< 0.001	0%, *P* = 0.99
1-year	5	RR: 6.38 (4.98–8.19)	< 0.001	58%, *P* = 0.05
Transient Ischemic Attack				
30-day	4	RR: 1.24 (0.45–3.42)	0.68	14%, *P* = 0.32
1-year	5	RR: 1.05 (0.61–1.81)	0.87	0%, *P* = 0.89
Major Vascular Complications				
30-day*	8	RR: 2.75 (2.31–3.27)	< 0.001	19%, *P* = 0.28
1-year*	4	RR: 1.46 (0.86–2.47)	0.16	35%, *P* = 0.20
Infective Endocarditis				
30-day	5	RR: 1.26 (0.46–2.47)	0.65	0%, *P* = 0.67
1-year	5	RR: 0.79 (0.33–1.85)	0.58	0%, *P* = 0.80
Atrial Fibrillation				
30-day	8	RR: 0.41 (0.22–0.76)	0.005	96%, *P* < 0.001
1-year	6	RR: 0.24 (0.17–0.33)	< 0.001	75%, *P* = 0.001
Acute Kidney Injury (Stage 2–3)*	5	RR: 0.33 (0.19–0.57)	< 0.001	0%, *P* = 0.69
Bleeding				
30-day	9	RR: 0.38 (0.21–0.70)	0.002	87%, *P* < 0.001
1-year*	4	RR: 0.33 (0.27–0.42)	< 0.001	0%, *P* = 0.67
Hospital Stay (days)	10	MD: −2.44 (−3.24 to −1.64)	< 0.001	93%, *P* < 0.001
Ejection Fraction (%)				
1-year	3	MD: 3.30 (−0.60 to 7.20)	0.10	95%, *P* < 0.001
1-year (Change)	3	MD: 3.41 (−0.88 to 7.70)	0.12	92%, *P* < 0.001
Aortic Valve Pressure Gradient (mmHg)				
30-day	8	MD: −1.39 (−3.13 to 0.35)	0.12	99%, *P* < 0.001
30-day (Change)*	5	MD: −2.83 (−3.67 to −1.98)	< 0.001	0%, *P* = 0.91
1-year	5	MD: −1.38 (−4.23 to 1.47)	0.34	99%, *P* < 0.001
1-year (Change)	3	MD: −1.00 (−3.97 to 1.79)	0.48	81%, *P* = 0.005
Aortic Valve Area (cm²)				
30-day	5	MD: 0.06 (−0.09 to 0.21)	0.44	97%, *P* < 0.001
30-day (Change)	4	MD: 0.12 (−0.11 to 0.36)	0.31	98%, *P* < 0.001

*CI* Confidence Interval, *I² *Heterogeneity statistic, *k* Number of studies, *MD* Mean Difference, *RR* Risk Ratio, *TAVI* Transcatheter Aortic Valve Implantation, *SAVR* Surgical Aortic Valve Replacement

* For these outcomes, the data presented are from a leave-one-out sensitivity analysis, performed because the primary analysis showed high and statistically significant heterogeneity. The number of studies (k) reflects the final analysis after removal of the influential study

### Mortality

#### Thirty-days mortality

Sixteen studies were pooled in a meta-analysis model of 30 days mortality [15, 16, 18, 20, 22–24, 26, 27, 31–33, 35–37, 42]. The pooled risk ratio favoured TAVI group over SAVR one (RR = 0.66; 95% CI, 0.5 to 0.76; *P* < 0.00001). Pooled studies showed no heterogeneity (*P* = 0.08; I² = 35%). A funnel plot was generated which showed minor visual imbalance, however, there is no clear absence of studies on one side of the RR which suggests the absence of publications bias regarding negative or neutral studies. Supplementary Fig. 1.

A subgroup analysis based on study design showed significantly lower 30 days mortality rate in TAVI group within the twelve pooled studies included in the subgroup of observational studies (RR = 0.65; 95% CI, 0.56 to 0.76; *P* = 0.02) [15, 16, 18, 20, 22–24, 26, 27, 32, 33, 42]. The subgroup showed heterogeneity between studies (*P* = 0.04; I² = 47%) which was resolved after removing Mehaffey et al. 2024 (*P* = 0.40; I² = 4%). The pooled RR after sensitivity analysis also favoured TAVI group (RR = 0.78; 95% CI, 0.63 to 0.97; *P* = 0.02).

**Fig. 3:**
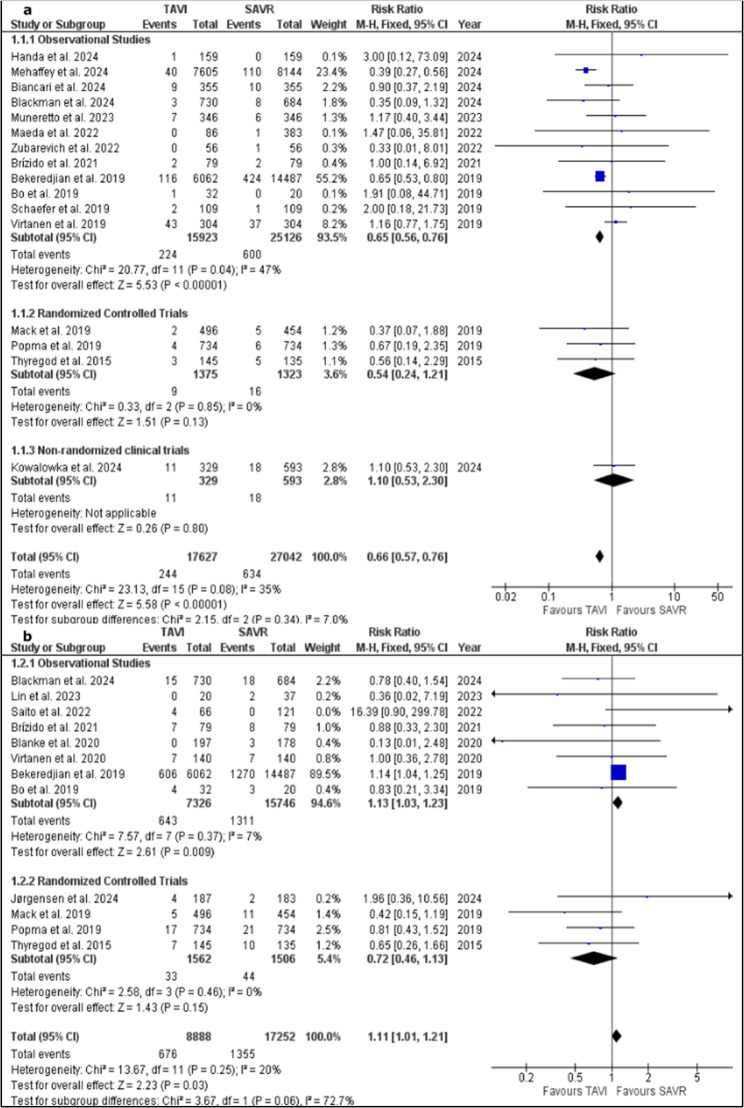
**a**: forest plot of 30-days events of mortality with a subgrouping based on study design and **b**: forest plot of one-year events of mortality with a subgrouping based on study design

On the other hand, in the subgroup of RCTs, the pooled RR of 3 studies did not favour either of the two groups (RR = 0.54; 95% CI, 0.24 to 1.22; *P* = 0.14) [35–37]. Heterogeneity between studies was resolved within the subgroup (*P* = 0.85; I² = 0%). The subgroup of non-randomized studies included only one study [31] which did not favour either of the two groups as well (RR = 1.10; 95% CI, 0.53 to 2.30; *P* = 0.80). Figure 3a.

#### One-year mortality

Twelve studies were included in the analysis of 1 year mortality [17, 21, 23, 25, 26, 30, 32, 34–37, 42]. The pooled risk ratio favoured SAVR group over TAVI one (RR = 1.11; 95% CI, 1.01 to 1.21; *P* = 0.03) Pooled studies showed no heterogeneity (*P* = 0.25; I² = 20%).

In a subgroup analysis based on study design, eight studies were pooled in the subgroup of observational studies [17, 21, 23, 25, 26, 30, 32, 42]. The pooled RR showed a significantly lower one-year mortality rate in SAVR group (RR = 1.13; 95% CI, 1.03 to 1.23; *P* = 0.009). The eight included studies showed homogeneity within the subgroup (*P* = 0.37; I² = 7%). A funnel plot was generated to explore the potential presence of publication bias. Visual asymmetry was evident which suggests small-study effects and a potential publication bias of studies that showed unfavourable one-year mortality outcome. Supplementary Fig. 2.

The pooled RR of the four studies included in the subgroup of RCTs did not favor either of the two groups (RR = 0.72; 95% CI, 0.46 to 1.13; *P* = 0.15). The four included studies showed homogeneity within the subgroup (*P* = 0.46; I² = 0%). Figure 3b.

#### Long-term mortality

Thirteen studies were included in a meta-analysis of long-term mortality (2–10 years) [15–17, 19, 24, 27, 28, 39, 40, 42–44]. The pooled risk ratio did not favour either of the two groups (RR = 1.10; 95% CI, 0.89 to 1.36; *P* = 0.36). Heterogeneity between studies was observed in the analysis model (*P* < 0.00001; I² = 78%). Heterogeneity was not resolved after performing sensitivity analysis by removing single study in each scenario. The visual overall symmetry of the generated funnel plot, with small studies distributed on both sides of the pooled RR, suggested the absence of publication bias. Supplementary Fig. 3.

In a subgroup analysis based on study design, the subgroup of observational studies included ten studies [15–17, 19, 24, 26–28, 42, 43]. The pooled RR did not favour either of the two groups (RR = 1.16; 95% CI, 0.87 to 1.54; *P* = 0.31). The ten included studies showed heterogeneity within the subgroup (*P* < 0.00001; I² = 82%) which remained high after removing one study in each scenario.

The pooled RR of the three studies included in the subgroup of RCTs did not favour either of the two groups as well (RR = 1.01; 95% CI, 0.84 to 1.22; *P* = 0.93) [39, 40, 44]. Heterogeneity was resolved within the subgroup of RCTs (*P* = 0.32; I² = 11%). Supplementary Fig. 4.

Subgroups were also constituted based on the point of time were mortality rates measured. Eight studies were included in the subgroup of 2 years mortality [17, 19, 26, 36, 40, 45–47]. The pooled RR did not favour either of the two groups (RR = 0.91; 95% CI, 0.69 to 1.20; *P* = 0.52). Pooled studies were homogenous (*P* = 0.07; I² = 46%).

Five studies were included in subgroup of 3 years mortality [6, 16, 21, 26, 28]. The pooled studies did not favour either of the two groups (RR = 1.00; 95% CI, 0.7 to 1.30; *P* = 0.98). Pooled studies were homogenous (*P* = 0.10; I² = 49%).

Three studies were pooled in the subgroup of 4 years mortality [42, 43, 48]. The pooled studies favoured TAVI group over SAVR one (RR = 0.6; 95% CI, 0.64 to 0.92; *P* = 0.004). Pooled studies were homogenous (*P* = 0.92; I² = 0%).

Four studies were pooled in the subgroup of 5 year mortality [15, 27, 38, 44]. The pooled studies did not favour either SAVR or TAVI groups (RR = 1.85; 95% CI, 0.99 to 3.46; *P* = 0.05). Pooled studies were heterogenous (*P* = 0.0005; I² = 83%) and sensitivity analysis could not resolve heterogeneity within the subgroup.

Only 1 study reported 6 years mortality which did not favour either of the groups (RR = 1.12; 95% CI, 0.84 to 1.50; *P* = 0.43) [47]. Additionally, 2 studies reported the outcome of 10 years mortality and their pooled risk ratio did not favour either of the two groups (RR = 1.13; 95% CI, 0.86 to 1.48; *P* = 0.38). Pooled studies were heterogenous (*P* = 0.03; I² = 78%) and sensitivity analysis could not resolve heterogeneity within the subgroup. A statistically significant difference was observed between subgroups (*P* = 0.02). Supplementary Fig. 5.

### Stroke

#### Thirty-days stroke

Thirteen studies were pooled in the meta-analysis model of 30 days stroke rate [15, 16, 18, 20, 22, 26, 27, 32, 33, 35–37, 42]. The pooled studies favoured TAVI group over SAVR one (RR = 0.70; 95% CI, 0.53 to 0.92; *P* = 0.01). Pooled studies were homogenous (*P* = 0.52; I² = 0%). The generated funnel plot showed no substantial asymmetry of studies on both sides of the RR. Supplementary Fig. 6.

In a subgroup analysis based on study design, ten studies were pooled in the subgroup of observational studies [15, 16, 18, 20, 22, 26, 27, 30, 32, 42]. The pooled RR showed a significantly lower 30 days stroke rate in TAVI group (RR = 0.71; 95% CI, 0.52 to 0.97; *P* = 0.03). The ten included studies showed homogeneity within the subgroup (*P* = 0.64; I² = 0%).

**Fig. 4:**
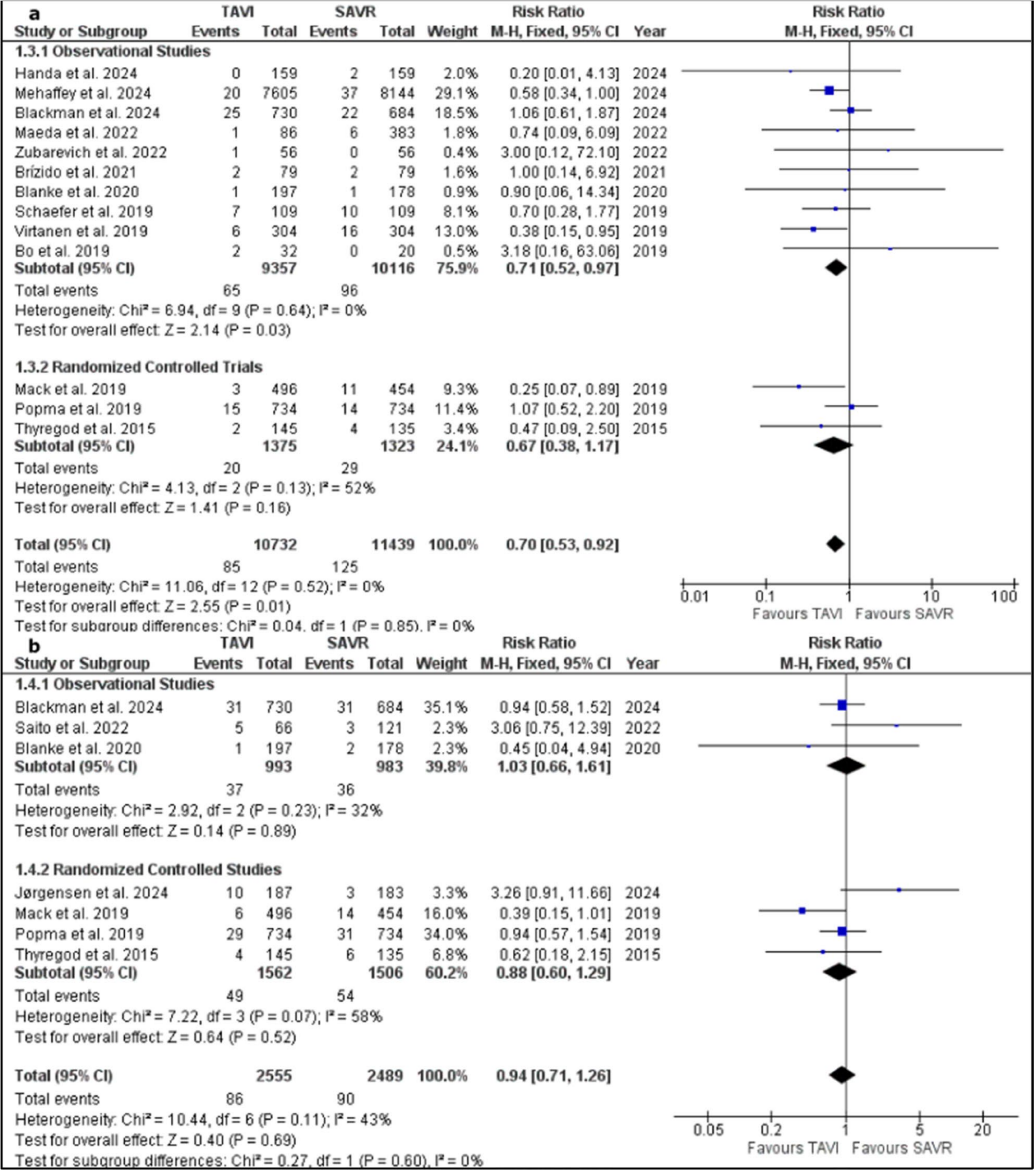
**a**: forest plot of 30-days events of stroke with a subgrouping based on study design and **b**: forest plot of one-year events of stroke with a subgrouping based on study design

The subgroup of RCTs included 3 studies [35–37] with a pooled RR that did not favour either of the two groups (RR = 0.67; 95% CI, 0.38 to 1.17; *P* = 0.16). The three included studies showed no heterogeneity within the subgroup as well (*P* = 0.13; I² = 52%). Figure 4a.

#### One-year stroke

Seven studies were pooled in the meta-analysis model of 1 year stroke rate [17, 30, 35–37, 39, 42]. The pooled studies favoured neither TAVI nor SAVR groups (RR = 0.94; 95% CI 0.71 to 1.26; *P* = 0.69). Pooled studies were homogenous (*P* = 0.11; I² = 43%).

In a subgroup analysis based on study design, the subgroup of observational studies included 3 studies [17, 30, 42]. The pooled RR of the subgroup showed no significant difference between TAVI and SAVR groups in terms of one-year stroke (RR = 1.03; 95% CI, 0.66 to 1.61; *P* = 0.89) The subgroup showed no heterogeneity between studies (*P* = 0.23; I² = 32%).

The subgroup of RCTs included 4 studies [34–37] with a pooled RR that showed no significant difference between the two groups as well (RR = 0.88; 95% CI, 0.60 to 1.26; *P* = 0.52). The four included studies showed heterogeneity within the subgroup (*P* = 0.07; I² = 58%) which was not resolved after removing one study in each scenario within the subgroup. Figure 4b.

#### Long-term stroke

Seven studies were also included in the model of long-term stroke rate (2–5 years) [17, 27, 38, 40, 42–44]. The pooled studies did not favour either of the two groups (RR = 0.88; 95% CI 0.72 to 1.07; *P* = 0.20). Pooled studies showed no heterogeneity (*P* = 0.19; I² = 31%).

In a subgroup analysis based on study design, the subgroup of observational studies included 4 studies [17, 27, 42, 43]. The pooled RR of the subgroup did not favour either TAVI or SAVR groups in terms of Long-term stroke (RR = 0.89; 95% CI, 0.71 to 1.13; *P* = 0.34). The subgroup showed no heterogeneity between studies (*P* = 0.18; I² = 39%).

The subgroup of RCTs included 3 studies [38, 40, 44] with a pooled RR that did not favour either of the two groups as well (RR = 0.83; 95% CI, 0.56 to 1.24; *P* = 0.36). The three included studies showed no heterogeneity within the subgroup (*P* = 0.15; I² = 47%). Supplementary Fig. 7.

### Permanent pacemaker implantation (PPI)

#### Thirty-days’ events of PPI

Thirteen studies were included in the meta-analysis model of events of permanent pacemaker implantation after 30 days of the procedure [15, 16, 18, 20, 22, 26, 27, 31, 33, 35–37, 42]. Pooled studies favoured SAVR group over TAVI group (RR = 2.44; 95% CI, 1.76 to 3.40; *P* < 0.00001). Pooled studies were heterogenous (*P* < 0.00001; I² = 75%). Sensitivity analysis did not resolve heterogeneity between pooled studies.

A subgroup analysis based on study design was carried out. Nine studies were included in the subgroup of observational design [15, 16, 18, 20, 22, 26, 27, 33, 42]. The pooled RR of the subgroup showed a significantly lower rate of PPI in SAVR group compared to TAVI group (RR = 2.55; 95% CI, 2.00 to 3.26; *P* < 0.00001). The included studies within the subgroup showed homogeneity (*P* = 0.14; I² = 35%).

The subgroup of RCTs included 3 studies [35–37] with a pooled RR that did not favour either of the two groups (RR = 3.12; 95% CI, 0.85 to 11.40; *P* = 0.09). Heterogeneity was observed between the three studies (*P* < 0.00001; I² = 94%). Additionally, one study was included in the subgroup of non-randomized studies [31]. This non-randomized study did not favour either of the two groups as well (RR = 1.47; 95% CI, 0.62 to 3.52; *P* = 0.38). Supplementary Fig. 8.

Another subgroup analysis was performed based on the route used for the procedure. Five studies were included in the subgroup of transfemoral approach [18, 20, 27, 31, 35] and 9 studies were included in the subgroup of other routes [15, 16, 22, 26, 33, 36, 37, 42]. The overall risk ratio of the transfemoral approach subgroup did not favour either of the two groups (RR = 1.62; 95% CI 0.84 to 3.10; *P* = 0.15) and pooled studies within the subgroup remained heterogenous (*P* = 0.01; I² = 68%). On the other hand, the overall risk ratio of the subgroup of other routes favoured SAVR group over TAVI group (RR = 2.93; 95% CI, 2.20 to 3.91; *P* < 0.00001 but pooled studies remained heterogenous (*P* = 0.02; I² = 75%). No significant difference was observed between groups (*P* = 0.10).

Heterogeneity was resolved within transfemoral route subgroup and other routes subgroup after removing Mack et al., 2019 and Thyregod et al., 2015 (*P* = 0.16; I² = 42% and *P* = 0.27; I² = 21% respectively). The overall risk ratio the transfemoral route subgroup favoured SAVR group over TAVI group (RR = 2.03; 95% CI 1.08 to 3.83; *P* = 0.03). Also, the overall risk ratio of the subgroup of other routes still favoured SAVR group over TAVI one (RR = 2.70; 95% CI, 2.26 to 3.23; *P* < 0.00001). No significant difference was observed between groups after sensitivity analysis (*P* = 0.40). Supplementary Fig. 9.

#### One-year’s events of PPI

Eight studies were pooled in the meta-analysis model of events of permanent pacemaker implantation after 1 year of the procedure [17, 21, 25, 34–37, 42]. Pooled studies favoured SAVR group over TAVI group (RR = 2.64; 95% CI 1.83 to 3.82; *P* < 0.00001). Pooled studies were heterogenous (*P* = 0.006; I² = 64%). Heterogeneity was resolved after removing Thyregod et al., 2015 from the meta-analysis model (*P* = 0.17; I² = 34%). After sensitivity analysis, the overall risk ratio still favoured SAVR group over TAVI one (RR = 2.33; 95% CI 1.80 to 3.02; *P* < 0.00001). Supplementary Fig. 10.

A subgroup analysis based on study design was conducted. Four studies were included in the subgroup of observational design [17, 21, 25, 42]. The pooled RR of the subgroup showed a significantly lower rate of PPI in SAVR group compared to TAVI group (RR = 2.70; 95% CI, 2.05 to 3.56; *P* < 0.00001). The subgroup showed homogeneity between studies (*P* = 0.75; I² = 0%).

The subgroup of RCTs included 4 studies as well [34–36, 38] with a pooled RR that favoured SAVR group (RR = 2.81; 95% CI, 1.41 to 5.58; *P* = 0.003). Heterogeneity was observed between studies (*P* = 0.0003; I² = 84%). Removing single study in each scenario did not resolve heterogeneity within the subgroup. Supplementary Fig. 11.

#### Valve thrombosis

Four studies were pooled in the meta-analysis model of events of valve thrombosis after 1 year [30, 34–36]. The overall risk ratio favoured neither TAVI nor SAVR groups (RR = 2.12; 95% CI 0.73 to 6.12; *P* = 0.17). Pooled studies were homogenous (*P* = 0.49; I² = 0%).

In a subgroup analysis based on study design, observational design subgroup included only 1 study [30], while RCTs subgroup included 3 studies [34–36]. The pooled RR of both subgroups did not favour either TAVI nor SAVR groups with (RR = 2.85; 95% CI, 0.12 to 69.49; *P* = 0.52) and (RR = 2.03; 95% CI, 0.66 to 6.28; *P* = 0.31). The subgroup of RCTs showed no heterogeneity between studies within the subgroup (*P* = 0.31; I² = 16%). No significant difference was observed between both subgroups. Supplementary Fig. 12.

### Aortic reintervention

#### Thirty-days’ events of aortic reintervention

Four studies were pooled in the meta-analysis model of events of aortic reintervention after 30 days [18, 22, 36, 42]. The overall risk ratio favoured TAVI group over SAVR group (RR = 0.35; 95% CI 0.13 to 0.92; *P* = 0.03). Pooled studies were homogenous (*P* = 0.45; I² = 0%).

In a subgroup analysis based on study design, observational design subgroup included 3 studies [18, 22, 42], while RCTs subgroup included only 1 study [36]. Observational design subgroup showed a lower rate of aortic reintervention in TAVI group after 30 days compared to SAVR group (RR = 0.27; 95% CI, 0.08 to 0.89; *P* = 0.03). On the other hand, the subgroup of RCTs showed no significant difference between the two groups (RR = 0.67; 95% CI, 0.11 to 3.98; *P* = 0.66). Observational design subgroup showed no heterogeneity between studies within the subgroup (*P* = 0.32; I² = 13%). No significant difference was observed between both subgroups. Supplementary Fig. 13.

#### One-year’s events of aortic reintervention

Four studies were pooled in the meta-analysis model of events of aortic reintervention after 1 year [34–36, 42]. The overall risk ratio did not favour either of the two groups (RR = 1.04; 95% CI 0.49 to 2.20; *P* = 0.93). Pooled studies were homogenous (*P* = 0.80; I² = 0%).

In a subgroup analysis based on study design, observational design subgroup included only 1 study [42], while RCTs subgroup included 3 studies. Both observational design and RCTs subgroups showed no significant difference between TAVI and SAVR groups in terms of aortic reintervention after 1 year of the procedure (RR = 1.25; 95% CI, 0.28 to 5.56; *P* = 0.77) and (RR = 0.97; 95% CI, 0.41 to 2.33; *P* = 0.95) for each subgroup respectively. RCTs subgroup showed no heterogeneity between studies within the subgroup (*P* = 0.63; I² = 0%). No significant difference was observed between both subgroups. Supplementary Fig. 14.

### Paravalvular leakage

#### Thirty-days’ events of paravalvular leakage

Four studies were pooled in the meta-analysis model of 30 days of events of paravalvular leakage after 30 days of the procedure [18, 20, 35, 36]. Pooled studies favoured SAVR group over TAVI group (RR = 5.60; 95% CI 2.98 to 10.53; *P* < 0.00001). Pooled studies were heterogenous (*P* = 0.007; I² = 75%). Heterogeneity was resolved after removing Virtanen et al., 2019 from the meta-analysis model (*P* = 0.99; I² = 0%). After sensitivity analysis, the overall risk ratio still favoured SAVR group over TAVI one (RR = 7.56; 95% CI 6.04 to 9.47; *P* < 0.00001). Supplementary Fig. 15.

In a subgroup analysis based on study design, observational design subgroup included 2 studies [18, 20] and RCTs subgroup included 2 studies as well [35, 36]. Both observational design and RCTs subgroups showed lower rates of paravalvular leakage in SAVR group after 30 days of the procedure compared to TAVI (RR = 4.58; 95% CI, 1.68 to 12.49; *P* = 0.003) and (RR = 7.53; 95% CI, 5.96 to 9.51; *P* < 0.00001) for each subgroup respectively. Heterogeneity was observed in the subgroup of observational studies (*P* = 0.03; I² = 78%), while RCTs subgroup showed homogeneity between studies within the subgroup (*P* = 0.98; I² = 0%) Supplementary Fig. 16.

#### One-year’s events of paravalvular leakage

Five studies were pooled in the meta-analysis model of the events paravalvular leakage after 1 year of the procedure [25, 27, 35–37]. The overall risk ratio favoured SAVR group over TAVI group (RR = 6.38; 95% CI 4.98 to 8.19; *P* < 0.00001). Pooled studies were homogenous (*P* = 0.05; I² = 58%).

In a subgroup analysis based on study design, observational design subgroup included 2 studies [25, 27] and RCTs subgroup included 3 studies [35–37]. Both observational design and RCTs subgroups showed lower rates of paravalvular leakage in SAVR group after 1 year of the procedure compared to TAVI (RR = 8.11; 95% CI, 4.44 to 14.79; *P* < 0.00001) and (RR = 6.05; 95% CI, 4.60 to 7.95; *P* < 0.00001) for each subgroup respectively. Observational design subgroup showed homogeneity between studies within the subgroup (*P* = 0.32; I² = 0%). On the other hand, heterogeneity was observed in the subgroup of RCTs (*P* = 0.02; I² = 74%). No significant difference was observed between both subgroups. Supplementary Fig. 17.

### Transient ischemic attack

#### Thirty-days’ events of transient ischemic attack

Four studies were pooled in the meta-analysis model of events transient ischemic attacks after 30 days [30, 35–37]. The overall risk ratio favoured neither TAVI nor SAVR group (RR = 1.24; 95% CI 0.45 to 3.42; *P* = 0.68). Pooled studies were homogenous (*P* = 0.32; I² = 14%).

In a subgroup analysis based on study design, observational design subgroup included only 1 study [30] and RCTs subgroup included 3 studies [35–37]. Both observational design and RCTs subgroups showed no significant difference between TAVI and SAVR groups (RR = 2.71; 95% CI, 0.11 to 66.15; *P* = 0.54) and (RR = 1.12; 95% CI, 0.38 to 3.29; *P* = 0.84) for each subgroup respectively. No heterogeneity was observed in the subgroup of RCTs (*P* = 0.19; I² = 40%). No significant difference was observed between both subgroups. Supplementary Fig. 18.

#### One-year’s events of transient ischemic attack

Five studies were pooled in the meta-analysis model of the events transient ischemic attacks after 1 year [30, 34–37]. The overall risk ratio favoured neither TAVI nor SAVR group (RR = 1.05; 95% CI 0.61 to 1.81; *P* = 0.87). Pooled studies were homogenous (*P* = 0.89; I² = 0%).

In a subgroup analysis based on study design, observational design subgroup included only 1 study [30] and RCTs subgroup included 3 studies [34–37]. Both observational design and RCTs subgroups showed no significant difference between TAVI and SAVR groups (RR = 2.71; 95% CI, 0.11 to 66.15; *P* = 0.54) and (RR = 1.01; 95% CI, 0.58 to 1.76; *P* = 0.86) for each subgroup respectively. No heterogeneity was observed in the subgroup of RCTs (*P* = 0.86; I² = 0%). No significant difference was observed between both subgroups. Supplementary Fig. 19.

### Major vascular complications

#### Thirty-days major vascular complications

Nine studies were pooled in the meta-analysis model of events of major vascular complications after 30 days of the procedure [15, 16, 18, 20, 33, 35–37, 42]. Pooled studies favoured SAVR group over TAVI group (RR = 2.43; 95% CI 1.74 to 3.39; *P* < 0.00001). Pooled studies were heterogenous (*P* < 0.0001; I² = 76%). Heterogeneity was resolved after removing Mack et al., 2019 from the meta-analysis model (*P* = 0.28; I² = 19%). After sensitivity analysis, the overall risk ratio still favoured SAVR group over TAVI one (RR = 2.75; 95% CI 2.31 to 3.27; *P* < 0.00001). Supplementary Fig. 20.

In a subgroup analysis based on study design, 6 studies were included in the subgroup of observational design [15, 16, 18, 20, 33, 42]. The pooled RR of the subgroup showed a significantly lower rate of major vascular complications in SAVR group compared to TAVI group (RR = 2.73; 95% CI, 2.12 to 3.52; *P* < 0.00001). The subgroup showed homogeneity between studies (*P* = 0.14; I² = 40%).

The subgroup of RCTs included 3 studies [35–37] with a pooled RR that did not favour either of the two groups (RR = 1.87; 95% CI, 0.66 to 5.31; *P* = 0.003). Heterogeneity was observed between studies (*P* < 0.0001; I² = 90%). Supplementary Fig. 21.

#### One-year major vascular complications

Five studies were pooled in the meta-analysis model of events of major vascular complications after 1 year of the procedure [17, 25, 35, 36, 42]. Pooled studies favoured SAVR group over TAVI group (RR = 1.95; 95% CI 1.07 to 3.55; *P* = 0.03). Pooled studies were heterogenous (*P* = 0.005; I² = 73%). Heterogeneity was resolved after removing Popma et al., 2019 from the meta-analysis model (*P* = 0.20; I² = 35%). After sensitivity analysis, the overall risk ratio did not favour either of the two groups (RR = 1.46; 95% CI 0.86 to 2.47; *P* = 0.16). Supplementary Fig. 22.

In a subgroup analysis based on study design, 3 studies were included in the subgroup of observational design [17, 25, 42]. The pooled RR of the subgroup showed no significant difference between the two groups in terms of major vascular complications (RR = 2.24; 95% CI, 0.60 to 8.37; *P* < 0.23). The subgroup showed homogeneity between studies (*P* = 0.10; I² = 57%). The subgroup of RCTs included 2 studies [35, 36] with a pooled RR that favoured SAVR group (RR = 2.05; 95% CI, 0.97 to 4.33; *P* = 0.06). Heterogeneity was observed between studies (*P* = 0.01; I² = 84%). Supplementary Fig. 23.

### Infective endocarditis

#### Thirty-days’ events of infective endocarditis

Five studies were pooled in the meta-analysis model of events of endocarditis after 30 days of the procedure [30, 33, 35–37]. The overall risk ratio favoured neither TAVI nor SAVR groups (RR = 1.26; 95% CI 0.46 to 2.47; *P* = 0.65). Pooled studies were homogenous (*P* = 0.67; I² = 0%).

A subgroup analysis based on study design was conducted. Two studies were included in the subgroup of observational design [30, 33]. The pooled RR of the subgroup showed no significant difference between the two groups (RR = 0.97; 95% CI, 0.22 to 4.21; *P* = 0.97). The subgroup of RCTs included 3 studies [35–37] with a pooled RR that showed no significant difference between the two groups as well (RR = 1.60; 95% CI, 0.39 to 6.61; *P* = 0.52). Each subgroup showed homogeneity among its included studies with (*P* = 0.39; I² = 0%) and (*P* = 0.49; I² = 0%) for the observational studies and RCTs subgroups, respectively. Supplementary Fig. 24.

#### One-year’s events of infective endocarditis

Five studies were also pooled in the meta-analysis model of events of endocarditis after 1 year of the procedure [30, 34–37]. The overall risk ratio did not favour either of the two groups (RR = 0.79; 95% CI 0.33 to 1.85; *P* = 0.58). Pooled studies were homogenous (*P* = 0.80; I² = 0%).

In a subgroup analysis based on study design, observational design subgroup included only one study [30] while RCTs subgroup included four studies [34–37]. Both observational design and RCTs subgroups showed no significant difference between TAVI and SAVR groups with (RR = 0.30; 95% CI, 0.01 to 7.35; *P* = 0.46) and (RR = 0.86; 95% CI, 0.35 to 2.12; *P* = 0.75) for each group respectively. The four studies included in the subgroup of RCTs showed no heterogeneity within the subgroup (*P* = 0.74; I² = 0%). Supplementary Fig. 25.

### Atrial fibrillation

#### Thiry-days’ events of atrial fibrillation

Eight studies were included in the meta-analysis model of events of atrial fibrillation after 30 days of the procedure [20, 26, 30, 33, 35–37, 42]. Pooled studies favoured TAVI group over SAVR group (RR = 0.41; 95% CI, 0.22 to 0.76; *P* = 0.005). Pooled studies were heterogenous (*P* < 0.00001; I² = 96%). Sensitivity analysis did not resolve heterogeneity between pooled studies.

In a subgroup analysis based on study design, observational design subgroup included five studies [20, 26, 30, 33, 42] while RCTs subgroup included three studies [35–37]. Observational design subgroup showed no significant difference between TAVI and SAVR groups with (RR = 0.68; 95% CI, 0.26 to 1.80; *P* = 0.44). On the other hand, the subgroup if RCTs showed a lower atrial fibrillation rate in TAVI group after 30 days of the procedure compared to SAVR group (RR = 0.20; 95% CI, 0.13 to 0.31; *P* < 0.00001). Both subgroups showed heterogeneity between included studies with (*P* < 0.00001; I² = 96%) and (*P* = 0.02; I² = 74%) for the observational studies and RCTs subgroups, respectively. Supplementary Fig. 26.

Another subgroup analysis was performed based on the route used for the procedure. Four studies were included in the subgroup of transfemoral approach [20, 30, 35, 42] and 5 studies were included in the subgroup of other routes [26, 33, 36, 37]. The overall risk ratio of the transfemoral approach subgroup favoured TAVI group over SAVR one (RR = 0.29; 95% CI 0.14 to 0.61; *P* = 0.001) and pooled studies within the subgroup remained heterogenous (*P* < 0.00001; I² = 94%). On the other hand, the overall risk ratio of the subgroup of other routes did not favour either of the two groups (RR = 0.41; 95% CI, 0.14 to 2.06; *P* = 0.37) and pooled studies remained heterogenous (P = < 0.00001; I² = 97%). No significant difference was observed between groups (*P* = 0.63).

Omitting Muneretto et al., 2023 from the subgroup of other routes resolved heterogeneity within the subgroup (*P* = 0.38; I² = 38%). After sensitivity analysis, the overall risk ratio favoured TAVI group over SAVR group (RR = 0.24; 95% CI, 0.20 to 0.30; *P* < 0.00001). Conversely, sensitivity analysis did not resolve heterogeneity within the subgroup of transfemoral route. No significant difference was observed between subgroups (*P* = 0.66). Supplementary Fig. 27.

#### One-year’s events of atrial fibrillation

Six studies were included in the meta-analysis model of events of atrial fibrillation after 1 year of the procedure [30, 34–37, 42]. Pooled studies favoured TAVI group over SAVR group (RR = 0.24; 95% CI 0.17 to 0.33; *P* < 0.00001). Pooled studies were heterogenous (*P* = 0.001; I² = 75%). Sensitivity analysis did not resolve heterogeneity between pooled studies.

In a subgroup analysis based on study design, observational design subgroup included 2 studies [30, 42] while RCTs subgroup included four studies [34–37]. Both observational design and RCTs subgroups showed a lower rate of atrial fibrillation in TAVI group after 1 year of the procedure compared to SAVR group (RR = 0.36; 95% CI, 0.14 to 0.94; *P* = 0.04) and (RR = 0.21; 95% CI, 0.13 to 0.32; *P* = 0.001) for each group respectively. Both subgroups showed heterogeneity between included studies with (*P* = 0.06; I² = 72%) and (*P* = 0.0009; I² = 82%) for the observational studies and RCTs subgroups, respectively. No significant difference was observed between both subgroups. Supplementary Fig. 28.

A subgroup analysis was performed based on the route used for the procedure. Four studies were included in the subgroup of transfemoral approach [30, 34, 35, 42] and 2 studies were included in the subgroup of other routes [36, 37]. The overall risk ratio of the transfemoral approach subgroup favoured TAVI group over SAVR group (RR = 0.20; 95% CI 0.12 to 0.35; *P* < 0.00001) and pooled studies within the subgroup remained heterogenous (*P* = 0.003; I² = 78%). Also, the overall risk ratio of the subgroup of other routes favoured TAVI group over SAVR group (RR = 0.29; 95% CI, 0.21 to 0.33; *P* < 0.00001), however, pooled studies were homogenous (*P* = 0.13; I² = 56%). No significant difference was observed between subgroups (*P* = 0.25). Supplementary Fig. 29.

#### Acute kidney injury (stage 2–3)

Six studies were pooled in the meta-analysis model of events of acute kidney injury (stage 2–3) after 30 days of the procedure [18, 20, 34–37]. Pooled studies favoured neither TAVI nor SAVR groups (RR = 0.63; 95% CI 0.14 to 2.71; *P* = 0.53). Pooled studies were heterogenous (*P* < 0.00001; I² = 85%). Heterogeneity was resolved after removing Schaefer et al., 2019 from the meta-analysis model (*P* = 0.69; I² = 0%). After sensitivity analysis, the overall risk ratio favoured TAVI group over SAVR one (RR = 0.33; 95% CI 0.19 to 0.57; *P* < 0.0001). Supplementary Fig. 30.

In a subgroup analysis based on study design, observational design subgroup included 2 studies [18, 20] while RCTs subgroup included 4 studies [34–37]. Observational design subgroup showed no significant difference between TAVI and SAVR groups (RR = 2.58; 95% CI, 0.05 to 146.07; *P* = 0.64). On the other hand, the subgroup of RCTs showed a lower rate of stage 2–3 acute kidney injury in TAVI group compared to SAVR group (RR = 0.30; 95% CI, 0.15 to 0.59; *P* = 0.0005). Observational design subgroup showed heterogeneity between included studies (*P* < 0.00001; I² = 96%), while RCTs subgroup showed homogeneity between studies within the subgroup (*P* = 0.57; I² = 0%). Supplementary Fig. 31.

### Bleeding

#### Thirty-days’ Events of Bleeding

Nine studies were included in the meta-analysis model of events of bleeding after 30 days of the procedure [15, 18, 21, 32, 33, 35–37, 42]. Pooled studies favoured TAVI group over SAVR group (RR = 0.38; 95% CI, 0.21 to 0.70; *P* = 0.002). Pooled studies were heterogenous (*P* < 0.00001; I² = 87%). Sensitivity analysis did not resolve heterogeneity between pooled studies.

**Fig. 5:**
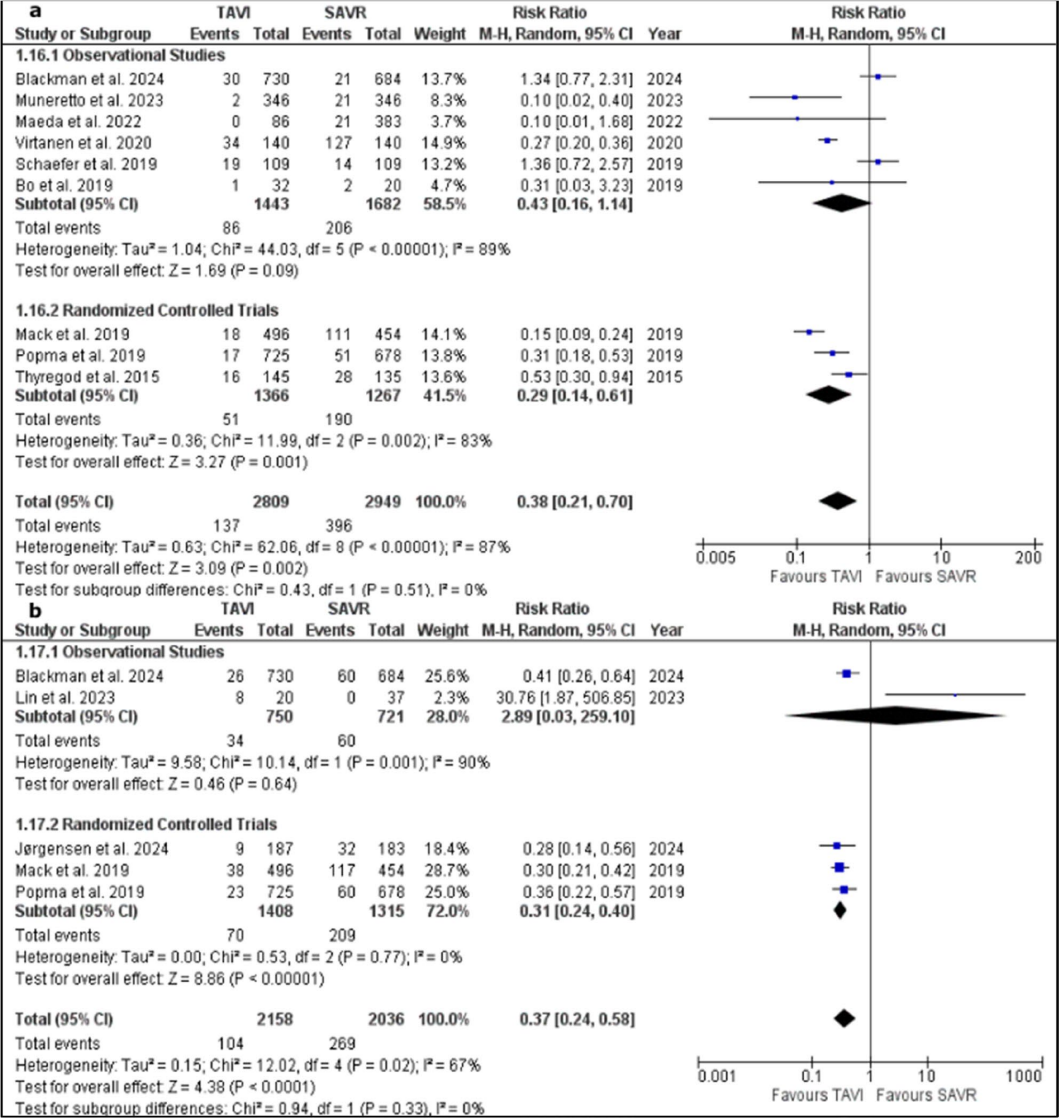
**a**: forest plot of 30-days events of bleeding with a subgrouping based on study design and **b**: forest plot of one-year events of bleeding with a subgrouping based on study design

In a subgroup analysis based on study design, observational design subgroup included 6 studies [15, 18, 21, 32, 33, 42] while RCTs subgroup included 3 studies [35, 36, 38]. Observational design subgroup showed no significant difference between TAVI and SAVR groups (RR = 0.43; 95% CI, 0.16 to 1.14; *P* = 0.09). On the other hand, the subgroup of RCTs showed a lower rate of bleeding in TAVI group after 30 days compared to SAVR group (RR = 0.29; 95% CI, 0.14 to 0.61; *P* = 0.001). Both subgroups showed heterogeneity between included studies with (*P* < 0.00001; I² = 89%) and (*P* = 0.002; I² = 83%) for the observational studies and RCTs subgroups, respectively. No significant difference was observed between both subgroups. Figure 5a.

Another subgroup analysis was performed based on the route used for the procedure. Four studies were included in the subgroup of transfemoral approach [18, 21, 35, 42] and 5 studies were included in the subgroup of other routes [15, 32, 33, 36, 37]. The overall risk ratio of the transfemoral approach subgroup did not favour either of the two groups (RR = 0.51; 95% CI, 0.19 to 1.39; *P* < 0.19) and pooled studies within the subgroup remained heterogenous (*P* < 0.00001; I² = 95%). On the other hand, the overall risk ratio of the subgroup of other routes favoured TAVI group over SAVR group (RR = 0.31; 95% CI, 0.17 to 0.56; *P* = 0.0001), however, pooled studies were homogenous (*P* = 0.16; I² = 39%). No significant difference was observed between subgroups (*P* = 0.42).

#### One-year’s events of bleeding

Five studies were pooled in the meta-analysis model of events of bleeding after 1 year of the procedure [25, 34–36, 42]. Pooled studies favoured TAVI group over SAVR group (RR = 0.37; 95% CI, 0.24 to 0.58; *P* < 0.0001). Pooled studies were heterogenous (*P* = 0.02; I² = 67%). Heterogeneity was resolved after removing Lin et al., 2023 from the meta-analysis model (*P* = 0.67; I² = 0%). After sensitivity analysis, the overall risk ratio still favoured TAVI group over SAVR one (RR = 0.33; 95% CI, 0.27 to 0.42; *P* < 0.00001).

In a subgroup analysis based on study design, observational design subgroup included 2 studies [25, 42] while RCTs subgroup included 3 studies [34–36]. Observational design subgroup showed no significant difference between TAVI and SAVR groups (RR = 2.89; 95% CI, 0.03 to 259.10; *P* = 0.64). On the other hand, the subgroup of RCTs showed a lower rate of bleeding in TAVI group after 1 year compared to SAVR group (RR = 0.31; 95% CI, 0.24 to 0.40; *P* < 0.00001). Observational design subgroup showed heterogeneity between included studies (*P* = 0.001; I² = 90%), while RCTs subgroup showed homogeneity between studies within the subgroup (*P* = 0.77; I² = 0%). No significant difference was observed between both subgroups. Figure 5b.

#### Hospital stay

Ten studies were included in a meta-analysis model that explored length of hospital stay in TAVI and SAVR groups [18, 20, 22, 25, 26, 28, 29, 32, 34, 35]. The pooled studies showed a significantly lower length of stay in TAVI group compared to SAVR one (MD = −2.44; 95% CI −3.24 to −1.64; *P* < 0.00001). Pooled studies were heterogenous (*P* < 0.00001; I² = 93%). Heterogeneity between studies were not resolved after removing single study in each scenario.

In a subgroup analysis based on study design, the subgroup of observational studies included 8 studies [18, 20, 22, 25, 26, 28, 29, 32]. The pooled MD of the subgroup showed a significantly lower length of stay in TAVI group compared to SAVR one (MD = −1.42; 95% CI −2.76 to −0.08; *P* = 0.04). The subgroup showed heterogeneity between studies (*P* < 0.00001; I² = 91%) which was not resolved by leave-one out test.

The subgroup of RCTs included only 2 studies [34, 35] with a pooled RR that favoured TAVI group as well (MD = −4.35; 95% CI, −4.49 to −4.21; *P* < 0.00001). The two included studies showed no heterogeneity within the subgroup (*P* = 0.93; I² = 0%). Supplementary Fig. 32.

Another subgroup analysis was performed based on the route used for the procedure. Seven studies were pooled in the subgroup of transfemoral approach [18, 20, 25, 28, 29, 34, 35]. The pooled studies favoured TAVI group over SAVR one (MD = −3.22; 95% CI −3.94 to −2.51; *P* < 0.00001). Pooled studies were heterogonous (*P* < 0.00001; I² = 91%) which was not resolved after removing one study from the subgroup in each scenario.

Three studies were included in the subgroup that included other approaches [22, 26, 32]. The pooled studies did not favour either of the two groups (MD = −0.85; 95% CI −2.12 to 0.43; *P* = 0.19). pooled studies were homogenous (*P* = 0.19; I² = 40%). Supplementary Fig. 33.

### Ejection fraction

#### One-year Ejection Fraction

Three studies were included in the meta-analysis model of ejection fraction after 1 year after the procedure [25, 27, 35]. The pooled studies favoured neither TAVI nor SAVR groups (MD = 3.30; 95% CI −0.60 to 7.20; *P* = 0.10). Pooled studies were heterogenous (*P* < 0.00001; I² = 95%).

In a subgroup analysis based on study design, the subgroup of observational studies included 2 studies [25, 27]. The pooled MD of the two studies favoured TAVI group (MD = 1.60; 95% CI 0.11 to 3.09; *P* = 0.03). The subgroup showed no heterogeneity between studies (*P* < 0.88; I² = 0%). The subgroup of RCTs included only 1 study [35] with a mean difference that favored TAVI group over SAVR group (MD = 6.40; 95% CI, 6.29 to 6.51; *P* < 0.00001). Supplementary Fig. 34.

#### Ejection fraction change

The meta-analysis model of change from baseline to 1 year after the procedure in ejection fraction included the same 3 studies [25, 27, 35]. The pooled studies did not favour either of the two groups (MD = 3.41; 95% CI −0.88 to 7.70; *P* = 0.12). Included studies were also heterogenous (*P* < 0.00001; I² = 92%).

In a subgroup analysis based on study design, the subgroup of observational studies included 2 studies [25, 27]. The pooled MD of the two studies did not favour either of the two groups (MD = 1.51; 95% CI, −1.52 to 4.55; *P* = 0.33). The subgroup showed no heterogeneity between studies (*P* = 0.10; I² = 63%).The subgroup of RCTs included only 1 study [35] with a mean difference that favoured TAVI group over SAVR group (MD = 6.90; 95% CI, 5.57 to 8.05; *P* < 0.00001). Supplementary Fig. 35.

### Aortic valve pressure gradient

#### Thirty-days pressure gradient

Eight studies were pooled in the meta-analysis model of pressure gradient after 30 days of the procedure [18, 22, 26, 30, 33, 35, 36, 40]. Pooled studies favoured neither TAVI nor SAVR groups (MD = −1.39; 95% CI −3.13 to 0.35; *P* = 0.12). Pooled studies were heterogenous (*P* < 0.00001; I² = 99%). Heterogeneity between studies remained unresolved after removing single study in each scenario.

In a subgroup analysis based on study design, the subgroup of observational studies included 5 studies [18, 22, 26, 30, 33]. The pooled MD of the subgroup favoured TAVI group over SAVR group (MD = −1.65; 95% CI, −3.13 to −0.16; *P* = 0.03). The subgroup showed heterogeneity between studies (*P* < 0.00001; I² = 93%) which was not resolved after leave-one out test.

The subgroup of RCTs included 3 studies [35, 36, 40] with a pooled MD that did not favour either of the two groups (MD = −0.89; 95% CI, −3.89 to 2.10; *P* = 0.56). Heterogeneity between studies was observed (*P* < 0.00001; I² = 100%) which was not resolved after leave-one out test. Supplementary Fig. 36.

Another subgroup analysis was performed based on the route used for the procedure. Five studies were included in the subgroup of transfemoral approach [18, 22, 30, 35, 40] and 3 st1udies were included in the subgroup of other approaches [26, 33, 36]. The overall mean difference of the transfemoral approach subgroup did not favour either of the two groups (MD = −0.77; 95% CI −2.92 to 1.39; *P* = 0.48) and pooled studies remained heterogenous within the subgroup (*P* < 0.00001; I² = 99%). On the other hand, the overall mean difference of the subgroup of other approaches favoured TAVI group over SAVR one (MD = −2.40; 95% CI −4.70 to −0.10; *P* = 0.04) but pooled studies remained heterogenous (*P* < 0.00001; I² = 96%). Supplementary Fig. 37.

#### Thirty-days pressure gradient change

Six studies were pooled in the meta-analysis model of change from baseline in valve pressure gradient after 30 days of the procedure [18, 25, 35–37, 40]. Pooled studies favoured TAVI group over SAVR group (MD = −1.85; 95% CI −3.42 to −0.28; *P* = 0.02). Pooled studies were heterogenous (*P* = 0.01; I² = 68%). Heterogeneity was resolved after removing Mack et al., 2019 from the meta-analysis model (*P* = − 0.91; I² = 0%). After sensitivity analysis, the overall mean difference still favoured TAVI group over SAVR one (MD = −2.83; 95% CI −3.67 to −1.98; *P* < 0.00001). Supplementary Fig. 38.

In a subgroup analysis based on study design, the subgroup of observational studies included 2 studies [18, 25]. The pooled MD of the two studies did not favour either of the two groups (MD = −1.47; 95% CI, −5.47 to 2.53; *P* = 0.47). The subgroup showed no heterogeneity between studies (*P* = 0.61; I² = 0%).

The subgroup of RCTs included 4 studies [35–37, 40] with a pooled MD that favoured TAVI group (MD = −1.92; 95% CI, −3.74 to −0.10; *P* = 0.04). Heterogeneity between studies was observed (*P* = 0.003; I² = 78%). Heterogeneity was best resolved within the subgroup after removing Mack et al. 2019 (*P* = 0.86; I² = 0%). After sensitivity analysis, the mean difference of RCTs subgroup still favoured TAVI group over SAVR group (MD = −2.89; 95% CI, −3.75 to −2.03; *P* < 0.00001). Supplementary Fig. 39.

#### One-year pressure gradient

Five studies were pooled in the meta-analysis model of pressure gradient after 1 year of the procedure [25, 27, 30, 35, 36]. Pooled studies favoured neither TAVI nor SAVR groups (MD = −1.38; 95% CI −4.23 to 1.47; *P* = 0.34). Pooled studies were heterogenous (*P* < 0.00001; I² = 99%). Heterogeneity between studies remained high after removing single study in each scenario.

In a subgroup analysis based on study design, the subgroup of observational studies included 3 studies [25, 27, 30]. The pooled MD of the subgroup favoured TAVI group over SAVR group (MD = −2.09; 95% CI, −3.89 to −0.28; *P* = 0.02). The three pooled studies showed heterogeneity (*P* = 0.006; I² = 80%).

The subgroup of RCTs included 2 studies [35, 36] with a pooled MD that did not favour either of the two groups (MD = −0.24; 95% CI, −4.84 to 4.37; *P* = 0.92). Heterogeneity was observed between the two studies (*P* < 0.00001; I² = 100%). Supplementary Fig. 40.

Additional subgroup analysis was performed based on the route used for the procedure. Four studies were included in the subgroup of transfemoral approach [25, 27, 30, 35] and 1 study was included in the subgroup of other approaches [36]. The overall mean difference of the transfemoral approach subgroup did not favour either of the two groups (MD = −1.05; 95% CI −4.07 to 1.98; *P* = 0.50) and pooled studies remained heterogenous within the subgroup (*P* < 0.00001; I² = 98%). On the other hand, the included study in the subgroup of other approaches favoured TAVI group over SAVR one (MD = −2.60; 95% CI −3.23 to −1.97; *P* < 0.00001). Supplementary Fig. 41.

#### One-year pressure gradient change

Three studies were pooled in the meta-analysis model of change from baseline in valve pressure gradient after 1 year of the procedure [27, 35, 36]. Pooled studies did not favour either of the two groups (MD = −1.00; 95% CI −3.97 to 1.79; *P* = 0.48). Pooled studies were heterogenous within the model (*P* = 0.005; I² = 81%).

A subgroup analysis based on study design was carried out. One study was included in the subgroup of observational design [27]. The pooled MD of the subgroup did not favour either of the two groups (MD = −1.00; 95% CI, −4.07 to 2.07; *P* = 0.51). The subgroup of RCTs included 2 studies [35, 36] with a pooled MD that did not favour either of the two groups as well (MD = −1.02; 95% CI, −5.04 to 3.00; *P* = 0.62). Heterogeneity was observed between the two studies (*P* = 0.001; I² = 91%). Supplementary Fig. 42.

### Aortic valve area

#### Thirty-days aortic valve area

Five studies were pooled in the meta-analysis model of valve area after 30 days of the procedure [18, 33, 35, 36, 40]. Pooled studies favoured neither TAVI nor SAVR groups (MD = 0.06; 95% CI −0.09 to 0.21; *P* = 0.44). Pooled studies were heterogenous (*P* < 0.00001; I² = 97%). Sensitivity analysis did not resolve heterogeneity between studies.

In a subgroup analysis based on study design, the subgroup of observational studies included 2 studies [18, 33]. The pooled MD of the subgroup did not favour either of the two groups (MD = 0.00; 95% CI, −0.06 to 0.06; *P* = 1.00). No heterogeneity was observed within the subgroup (*P* = 1.00; I² = 0%).

The subgroup of RCTs included 3 studies [35, 36, 40] with a pooled MD that did not favour either of the two groups as well (MD = 0.10; 95% CI, −0.14 to 0.34; *P* = 0.42). Heterogeneity was observed between the two studies (*P* < 0.00001; I² = 97%). Supplementary Fig. 43.

Another subgroup analysis was performed based on the route used for the procedure. Three studies were included in the subgroup of transfemoral approach [18, 35, 40] and 2 studies were included in the subgroup of other routes [33, 36]. The overall mean difference of the transfemoral approach subgroup did not favour either of the two groups (MD = 0.03; 95% CI −0.19 to 0.25; *P* = 0.77) and pooled studies remained heterogenous within the subgroup (*P* < 0.00001; I² = 97%). Also, the overall mean difference of the subgroup of other routes did not favour either of the two groups (MD = 0.10; 95% CI −0.10 to 0.30; *P* = 0.32) and pooled studies remained heterogenous (*P* < 0.0001; I² = 94%). No significant difference was observed between subgroups (*P* = 0.65). Supplementary Fig. 44.

#### Thirty-days aortic valve area change

Four studies were included in the meta-analysis model of change from baseline in valve area after 30 days of the procedure [18, 35–37]. Pooled studies favoured neither TAVI nor SAVR groups (MD = 0.12; 95% CI −0.11 to 0.36; *P* = 0.31). Pooled studies were heterogenous (*P* < 0.00001; I² = 98%). Removing single study in each scenario did not resolve heterogeneity in any of the potential scenarios.

In a subgroup analysis based on study design, the subgroup of observational design included only 1 study [18]. The pooled MD of the subgroup did not favour either of the two groups (MD = 0.00; 95% CI, −0.15 to 0.15; *P* = 1.00). The subgroup of RCTs included 3 studies [35–37] with a pooled MD that did not favour either of the two groups (MD = 0.16; 95% CI, −0.13 to 0.46; *P* = 0.28). Heterogeneity was observed between the three studies (*P* < 0.00001; I² = 98%). Supplementary Fig. 45.

Another subgroup analysis was performed based on the route used for the procedure. Two studies were included in the subgroup of transfemoral approach [18, 35] and 2 studies were included in the subgroup of other routes [36, 37]. The overall mean difference of the transfemoral approach subgroup did not favour either of the two groups (MD = −0.08; 95% CI −0.16 to 0.01; *P* = 0.07) but pooled studies within the subgroup were homogenous (*P* = 0.18; I² = 44%). On the other hand, the overall mean difference of the subgroup of other routes favoured TAVI group over SAVR group (MD = 0.29; 95% CI, 0.10 to 0.49; *P* = 0.003) but pooled studies remained heterogenous (*P* = 0.005; I² = 88%). A significant difference was observed between groups (*P* = 0.0006). Supplementary Fig. 46.

## Discussion

This study represents the most comprehensive meta-analysis to date, synthesizing data from 30 studies and including 48,210 low-risk patients with severe aortic stenosis comparing transcatheter and surgical aortic valve replacement. Our study demonstrated that TAVR was associated with a significantly lower risk of all-cause mortality and stroke during the initial 30-day post-procedural period. However, these early survival and safety advantages were attenuated over time, with our overall analysis showing a benefit for SAVR at one year for mortality and an ultimate convergence toward equipoise in long-term follow-up for both outcomes. This early benefit of TAVR is further supported by a profound and durable reduction in major bleeding and new-onset atrial fibrillation. Conversely, these advantages are counterbalanced by a persistently and significantly higher risk of permanent pacemaker implantation (PPI) and paravalvular leakage (PVL) with TAVR.

The therapeutic landscape for severe aortic stenosis has been fundamentally altered by the widespread adoption of TAVR in recent years. While current European and American guidelines now endorse TAVR as the standard of care for patients at elevated surgical risk, SAVR is still recommended for younger, lower-risk individuals [49]. This crucial distinction hinges on the proven, multi-decade track record of surgical valves, which contrasts with the pivotal knowledge gap concerning the long-term durability of transcatheter bio-prostheses and its implications for a patient’s lifetime management of their valve disease.

Our meta-analysis aims to synthesize the most current evidence to help inform this evolving clinical dilemma.

### Mortality and stroke

Our analysis, supported by high-certainty evidence from randomized trials, indicated that TAVR was associated with a 34% reduction in the risk of all-cause mortality during the 30-day post-procedure follow-up compared with SAVR. Conversely, the apparent survival benefit for SAVR at one year was driven largely by observational data of lower certainty. This finding aligns with a consistent body of evidence, including meta-analyses by Khan et al. (2020) (RR 0.59, 95% CI: 0.38–0.92, *P* = 0.02) [50] and the RCT-only analyses by Chen et al. (2024) (RR 0.44, 95% CI: 0.20–0.98, *P* = 0.04) [51] and Kazemian et al. (2024), who reported a similar hazard ratio (HR 0.45, 95% CI: 0.26–0.77) [52]. However, this advantage was not sustained in our overall analysis, which, driven by observational data, favored SAVR at one year. However, this finding should be interpreted with caution, given its nominal statistical significance (*P* = 0.03) in the context of multiple comparisons and the potential for residual confounding in the observational cohorts. This contrasts sharply with recent RCT-only meta-analyses. Kazemian et al. (2024) found a continued mortality benefit for TAVR through the first year (HR 0.55, 95% CI: 0.37–0.81), after which the risk converged and became similar to SAVR (HR 0.95, 95% CI: 0.74–1.21) [52]. Similarly, Rahman et al. (2024) reported a strong one-year survival benefit for TAVR (RR 0.62, 95% CI: 0.46–0.82) that disappeared at intermediate-term follow-up (RR 0.95, 95% CI: 0.73–1.24). This discrepancy highlights the critical impact of study design on pooled estimates.

Similarly, for stroke, our findings indicated that TAVR was associated with a 30% reduced risk at 30 days. While some analyses showed only a non-significant trend (Khan et al., RR 0.64, 95% CI: 0.38–1.09), Kazemian et al. specified that this benefit was most pronounced for disabling stroke (OR 0.37, 95% CI: 0.18–0.75). Our observation that this benefit is transient, with equipoise at one year and beyond, is strongly supported by the long-term data from Chen et al. [51], Reddy et al. [53], and Kazemian et al. [52].

### Procedural morbidity and safety Trade-offs

Our analysis confirms that the less-invasive nature of TAVR translates into profound and durable reductions in key periprocedural morbidities, most notably new-onset atrial fibrillation (NOAF) and major bleeding. We found that TAVR was associated with a 59% relative risk reduction in 30-day NOAF, an effect even more pronounced in our RCT-only subgroup (RR = 0.20; *P* < 0.00001). This is strongly confirmed by Chen et al., who reported a similarly large risk reduction (RR 0.21, 95% CI: 0.14–0.31) [51]. However, Kazemian et al. introduced a critical long-term caveat, finding a significantly higher risk of new-onset AF with TAVR beyond one year (OR 2.56, 95% CI: 1.68–3.90) [52], suggesting different underlying mechanisms for late-onset versus acute perioperative AF. This benefit shares a common mechanistic basis with the equally impressive reduction in major bleeding complications. Our data showed TAVR significantly decreased bleeding events at 30 days, aligning perfectly with the results from Chen et al. (RR 0.29, 95% CI: 0.14–0.61) [51]. Both benefits are directly attributable to TAVR avoiding the primary triggers of surgical morbidity: sternotomy, extensive tissue dissection, and cardiopulmonary bypass.

### Device-related complications

The early safety advantages of TAVR are counterbalanced by a significantly higher risk of device-related complications. TAVR was associated with a persistent, more than two-fold increase in the risk of PPI at both 30 days and one year. This finding is highly consistent with the literature, with recent meta-analyses reporting a more than threefold increase in 30-day PPI risk (Khan et al., 2020: RR 3.30, 95% CI: 2.04–5.33; Chen et al., 2024: RR 3.59, 95% CI: 1.43–9.03) [50, 51]. Rhythm disturbances after TAVR are primarily caused by mechanical stress on the atrioventricular conduction system, and the high heterogeneity observed in our analysis (I²=75%) and others is largely explained by the differential risk between valve types. Furthermore, a critical finding is the significantly higher risk of paravalvular leakage (PVL), with a more than 5-fold increased risk at 30 days and one year. Mechanistically, this difference stems from the fundamental distinction between a surgically sutured valve, which provides a direct anatomical seal, and a transcatheter valve, which relies on radial force to prevent leakage. While newer-generation TAVR devices have significantly reduced moderate-to-severe PVL, our findings confirm that a residual risk persists. However, it is important to recognize that these pooled estimates represent an average effect across various valve generations. As TAVI technology continues to evolve with improved sealing skirts and delivery systems, the rates of PPI and PVL in clinical practice may be lower than those observed in this cumulative analysis. This is clinically paramount, as even mild PVL is linked to adverse outcomes. Unlike the transient benefits in mortality and stroke, the increased risks of PPI and PVL do not attenuate over time, representing clear long-term advantages for SAVR.

### Valve reintervention and durability

Our analysis of aortic reintervention reveals equipoise between TAVR and SAVR after the first 30 days. We found no difference in reintervention rates at one year, a finding consistent recent meta-analysis. For Instance, Rahman et al. reported no significant difference in valve reintervention at one-year (RR 1.63, 95% CI: 0.82–3.24) or intermediate-term follow-up (RR 0.97, 95% CI: 0.56–1.69) [54]. While these comparable short-to-intermediate term reintervention rates are reassuring, they do not yet address the pivotal question of long-term structural valve deterioration, which remains the most critical unknown and a key factor in the lifetime management of younger, low-risk patients.

Finally, it is important to acknowledge that the recruitment period of several included studies overlapped with the COVID-19 pandemic. While the lack of stratified data in the primary trials precluded a formal subgroup analysis, emerging evidence suggests that the pandemic did not negatively impact the safety or efficacy of TAVI. Recent research has demonstrated that despite logistical challenges—such as significantly longer times from diagnosis to procedure and shorter ICU stays—30-day mortality and procedural complication rates remained similar between the pre-pandemic and pandemic eras (Ref). This suggests that the inclusion of studies conducted during this period is unlikely to have introduced significant bias into our pooled safety estimates [55].

### Strengths of this study

This study possesses several key strengths. Firstly, its exceptionally large pooled sample size of 48,210 patients provides robust statistical power for assessing multiple outcomes, making it one of the largest syntheses on this topic to date. Secondly, the inclusion of both RCTs and observational studies offers a unique dual perspective, reflecting both the efficacy in an ideal trial setting and the effectiveness in real-world practice. Thirdly, our comprehensive time-stratified and subgroup analyses allow for a granular exploration of outcomes, highlighting the temporal nature of risks and the impact of study design on pooled results. Finally, our work provides a formal meta-analytic estimate for PVL, addressing a key evidence gap in recent reviews.

### Limitations

Despite its strengths, our study has several important limitations that warrant consideration. First, as a study-level meta-analysis, we were constrained by the aggregate data reported in the primary publications. The absence of individual patient data prevented us from performing granular subgroup analyses to explore the impact of crucial variables such as patient age, sex, bicuspid versus tricuspid anatomy, or specific comorbidities on outcomes. Second, the included studies span a period of rapid technological evolution in TAVR. Our analysis pools outcomes across various valve types and generations, meaning our pooled estimates represent an average effect that may not fully reflect the improved safety and performance of the latest-generation devices. Third, while our total sample size is large, the number of patients contributing to long-term follow-up is substantially smaller. Finally, the certainty of our conclusions regarding outcomes beyond five years is limited by the sparse data, as only a few of the included trials have reported mature, long-term results. Fourth, substantial statistical heterogeneity (I² > 50%) was observed across several secondary outcomes, including atrial fibrillation, major bleeding, acute kidney injury, and length of stay. While we attempted to explore the sources of this heterogeneity through sensitivity analyses and subgroup stratifications based on study design (RCT vs. observational) and follow-up duration, the heterogeneity often persisted. This likely reflects intrinsic differences in clinical practice across the included centers, such as varying protocols for postoperative rhythm monitoring, differing thresholds for transfusion or discharge, and the evolution of VARC definitions over the study period. Consequently, these pooled estimates should be interpreted as an average effect across diverse clinical settings rather than a precise prediction for a specific institution.

### Future directions

Our analysis highlights three critical areas for future research. First, extended follow-up (> 10 years) from the pivotal low-risk RCTs is essential to definitively assess long-term valve durability. Second, dedicated studies must focus on mitigating TAVR’s primary drawbacks—permanent pacemaker implantation and paravalvular leak—through refined techniques and next-generation devices. These points underscore the need to formally evaluate “lifetime management” strategies, including the safety and efficacy of sequential valve interventions in younger, low-risk patients. Finally, while this meta-analysis focused on clinical and hemodynamic outcomes, future studies should prioritize the systematic evaluation of patient-reported Quality of Life (QoL), as recent evidence highlights this as a pivotal factor in the decision-making process for younger, low-risk patients [56].

## Conclusions

In this comprehensive meta-analysis of low-risk patients, TAVI was associated with a lower risk of stroke, major bleeding, and atrial fibrillation in the first 30 days post-procedure compared with SAVR. TAVI also showed a lower risk of 30-day all-cause mortality in the overall pooled analysis; however, this finding was driven primarily by observational data and was not statistically significant in the RCT subgroup. Similarly, while aggregate data favored SAVR for mortality at one year, this result was influenced by observational studies, whereas RCTs demonstrated no significant difference. Regarding long-term follow-up (> 2 years), while survival rates appear comparable, these findings are currently limited by sparse data and wider confidence intervals. The procedural advantages of TAVI are counterbalanced by a significantly and persistently higher risk of permanent pacemaker implantation and paravalvular leakage. Consequently, the choice of intervention must be individualized within a multidisciplinary Heart Team, carefully balancing the early recovery benefits of TAVI against long-term device risks.

## Supplementary Information

Supplementary Material 1.

## Data Availability

Data available within the article or its supplementary materials.

## Abbreviations

AS: Aortic stenosis
TAVI: Transcatheter aortic valve implantation
SAVR: Surgical aortic valve replacement
RCTs: Randomized controlled trials
RR: Relative risk
CI: Confidence interval
STS: Society of thoracic surgeons
PRISMA: Preferred reporting items for systematic reviews and meta-analyses
NOS: Newcastle-Ottawa scale
ROB2: Risk of bias 2
GRADE: Grading of recommendations assessment, development and evaluation
WOS: Web of science
NYHA: New York heart association
AF: Atrial fibrillation
AVA: Aortic valve area
MD: Mean difference
PPI: Permanent pacemaker implantation
PVL: Paravalvular leakage
HR: Hazard ratio
OR: Odds ratio
NOAF: New-onset atrial fibrillation
