# Supplementary material for: Transcatheter versus surgical aortic valve replacement in low-risk patients with severe aortic stenosis: a systematic review and meta-analysis
Source: BMC Cardiovasc Disord. 2026 Feb 19;26:181. doi: 10.1186/s12872-026-05558-6 (PMC12930610; doi:10.1186/s12872-026-05558-6)
Supplement: Supplementary file 1 — Supplementary Material 1. [file 12872_2026_5558_MOESM1_ESM.docx]

Table 1. New-castle Ottawa assessment of the included cohort studies

| Study | Year | Selection | | | | Comparability | Outcome | | | Quality |
| --- | --- | --- | --- | --- | --- | --- | --- | --- | --- | --- |
|  |  | Representativeness of the exposed cohort | Selection of the non-exposed cohort | Ascertainment of exposure | Begin without outcome present |  | Outcome assessment | Follow-up length | Adequacy of outcome |  |
| Maeda et al. | 2022 | * | * | * | * | ** | * | * | * | Good |
| Mehaffey et al. | 2024 |  | * | * | * | ** | * | * |  | Good |
| Saito et al. | 2022 |  |  | * | * |  | * | * |  | Poor |
| Schaefer et al. | 2019 |  |  | * | * | * * | * | * |  | Fair |
| Vilalta et al. | 2021 | * | * | * | * | * * | * | * |  | Good |
| Virtanen et al. | 2019 | * | * | * | * | * * | * | * |  | Good |
| Virtanen et al. | 2020 | * | * | * | * | * * | * | * |  | Good |
| Zubarevich et al. | 2022 | * | * | * | * | * | * | * |  | Good |
| Bekeredjian et al. | 2019 | * | * | * | * | * * | * | * |  | Good |
| Biancari et al. | 2024 |  | * | * | * | * * | * | * |  | Good |
| Lin et al. | 2023 |  | * | * | * | * * | * | * |  | Good |
| Brizido et al. | 2021 | * | * | * | * | * | * | * |  | Good |
| Handa et al. | 2024 | * | * |  |  | * * |  | * | * | Fair |
| Kolar et al. | 2022 |  |  |  |  |  |  |  |  | poor |
| Chung et al. | 2023 | * | * | * | * | * |  | * | * | Good |
| Blanke et al. | 2020 | * | * | * | * | * * | * | * |  | Good |
| Kowalowka et al. | 2024 | * | * | * | * | ** | * | * | * | Good |
| Bo et al. | 2019 | * | * | * | * | ** | * | * | * | Good |

Table 2. New-castle Ottawa assessment of the case-control studies

| Study | Year | Selection | | | | Comparability | Exposure | | | Quality |
| --- | --- | --- | --- | --- | --- | --- | --- | --- | --- | --- |
|  |  | Adequate case definition | Representativeness of the cases | Selection of Controls | Definition of Controls |  | Ascertainment of exposure | Same ascertainment method for cases and controls | Non-Response rate |  |
| Muneretto et al. | 2023 |  | * | * | * | * * | * | * | * | Good |

The GRADE quality assessment approach indicated that the quality of our evidence-based results is High to Very low.

Table 1 shows a summary of the evidence's quality, the degree of the effect, and the source of information used in the estimated risk.

**Table 3. The quality of evidence as assessed by GRADE approach**

| **Certainty assessment** | | | | | | | **Certainty** |
| --- | --- | --- | --- | --- | --- | --- | --- |
| **№ of studies** | **Study design** | **Risk of bias** | **Inconsistency** | **Indirectness** | **Imprecision** | **Other considerations** |  |
| **Thirty days mortality** | | | | | | | |
| 14 | 10 cohort studies and 4 RCTs | Not serious | Not serious | Not serious | Not serious | None | ⨁⨁ Low |
| One year mortality | | | | | | | |
| 12 | 8 cohort studies and 4 RCTs | Not serious | Not serious | Not serious | Not serious | None | ⨁⨁ Low |
| **Long-term mortality** | | | | | | | |
| 13 | 10 cohort studies and 3 RCTs | Not serious | Serious^a^ | Not serious | Not serious | None | ⨁ Very low |
| **Thirty days stoke rate** | | | | | | | |
| 13 | 10 cohort studies and 3 RCTs | Not serious | Not serious | Not serious | Not Serious | None | ⨁⨁ Low |
| **One year stoke rate** | | | | | | | |
| 8 | 4 cohort studies and 4 RCTs^b^ | Not serious | Serious^c^ | Not serious | Not Serious | None | ⨁⨁⨁ Moderate |
| **Long-term stroke rate (2-5 years)** | | | | | | | |
| 7 | 4 cohort studies and 3 RCTs | Not serious | Not serious | Not serious | Not serious | None | ⨁⨁ Low |
| Permanent pacemaker implantation at 30 days | | | | | | | |
| 13 | 8 cohort studies, 3 RCTs, 1 case-control study and 1 non-randomized trial | Not serious | Serious^d^ | Not serious | Serious | None | ⨁ Very low |
| Permanent pacemaker implantation at 1 year | | | | | | | |
| 8 | 4 cohort studies and 4 RCTs^b^ | Not serious | Not serious^e^ | Not serious | Not serious | None | ⨁⨁⨁⨁ High |
| Major vascular complications at 30 days | | | | | | | |
| 9 | 6 cohort studies and 3 RCTs | Not serious | Not serious^f^ | Not serious | Not serious | None | ⨁⨁ Low |
| Major vascular complications at 1 year | | | | | | | |
| 5 | 3 cohort studies and 2 RCTs | Not serious | Not serious^g^ | Not serious | Not serious | None | ⨁⨁ Low |
| **Infective endocarditis at 30 days** I | | | | | | | |
| 5 | 3 RCTs and 2 cohort studies | Not serious | Not serious | Not serious | Seriousʰ | None | ⨁⨁⨁ Moderate |
| **Infective endocarditis at 1 year** I | | | | | | | |
| 5 | 4 RCTs and 1 cohort study | Not serious | Not serious | Not serious | Not serious | None | ⨁⨁⨁⨁ High |
| **Atrial Fibrillation at 30 days** | | | | | | | |
| 8 | 5 cohort studies and 3 RCTs | Not serious | Seriousⁱ | Not serious | Not serious | None | ⨁ Very low |
| **Atrial Fibrillation at 1 year** | | | | | | | |
| 6 | 4 RCTs and 2 cohort study | Not serious | Seriousʲ | Not serious | Not serious | None | ⨁⨁⨁ Moderate |
| **Stage 2-3 acute kidney injury at 30 days** | | | | | | | |
| 6 | 4 RCTs and 2 cohort study | Not serious | Not seriousᵏ | Not serious | Seriousˡ | None | ⨁⨁⨁ Moderate |
| **Length of hospital stay** | | | | | | | |
| 10 | 8 cohort studies and 2 RCTs | Not serious | Seriousᵐ | Not serious | Not serious | None | ⨁ Very low |
| **Bleeding after 30 days** | | | | | | | |
| 9 | 6 cohort studies and 3 RCTs | Not serious | Seriousⁿ | Not serious | Not serious | None | ⨁ Very low |
| **Bleeding after 1 year** | | | | | | | |
| 5 | 3 RCTs and 2 cohort studies | Not serious | Seriousᵒ | Not serious | Not serious | None | ⨁⨁⨁ Moderate |
| **Valve thrombosis at 30 days** | | | | | | | |
| 4 | 3 RCTs and 1 cohort study | Not serious | Not serious | Not serious | Seriousᵖ | None | ⨁⨁⨁ Moderate |
| **Aortic reintervention at 30 days** | | | | | | | |
| 4 | 3 cohort studies and 1 RCT | Not serious | Not serious | Not serious | Not serious | None | ⨁⨁ Low |
| **Aortic reintervention at 1 year** | | | | | | | |
| 4 | 3 RCTs and 1 cohort study | Not serious | Not serious | Not serious | Not serious | None | ⨁⨁ Low |
| **Paravalvular leakage at 30 days** | | | | | | | |
| 4 | 2 RCTs and 2 cohort studies^b^ | Not serious | Not serious^q^ | Not serious | Not seriousʳ | None | ⨁⨁⨁⨁ High |
| **Paravalvular leakage at 1 year** | | | | | | | |
| 5 | 3 RCTs and 2 cohort studies | Not serious | Not serious | Not serious | Not serious | None | ⨁⨁⨁⨁ High |
| **Transient ischemic attack at 30 days** | | | | | | | |
| 4 | 3 RCTs and 1 cohort study | Not serious | Not serious | Not serious | Seriousˢ | None | ⨁⨁⨁ Moderate |
| **Transient ischemic attack at 1 year** | | | | | | | |
| 5 | 4 RCTs and 1 cohort study | Not serious | Not serious | Not serious | Not serious | None | ⨁⨁⨁⨁ High |
| **Ejection fraction after 1 year** | | | | | | | |
| 3 | 2 cohort studies and 1 RCT | Not serious | Seriousᵗ | Not serious | Seriousᵘ | None | ⨁ Very low |
| **Ejection fraction change after 1 year** | | | | | | | |
| 3 | 2 cohort studies and 1 RCT | Not serious | Seriousᵛ | Not serious | Seriousʷ | None | ⨁ Very low |
| **Aortic valve gradient after 30 days** | | | | | | | |
| 8 | 5 cohort studies and 3 RCT | Not serious | Seriousˣ | Not serious | Seriousʸ | None | ⨁ Very low |
| **Aortic valve gradient change after 30 days** | | | | | | | |
| 6 | 4 RCTs and 2 cohort studies | Not serious | Not seriousᶻ | Not serious | Not serious | None | ⨁⨁⨁ Moderate |
| **Aortic valve gradient after 1 year** | | | | | | | |
| 5 | 3 cohort studies and 2 RCTs | Not serious | Seriousᵃᵃ | Not serious | Seriousᵇᵇ | None | ⨁ Very low |
| **Aortic valve gradient change after 1 year** | | | | | | | |
| 3 | 2 RCTs and 1 cohort study | Not serious | Seriousᶜᶜ | Not serious | Seriousᵈᵈ | None | ⨁⨁ Low |
| **Aortic valve area after 30 days** | | | | | | | |
| 5 | 3 RCTs and 2 cohort study | Not serious | Seriousᵉᵉ | Not serious | Not serious | None | ⨁⨁⨁ Moderate |
| **Aortic valve area change after 30 days** | | | | | | | |
| 4 | 3 RCTs and 1 cohort study | Not serious | Seriousᶠᶠ | Not serious | Not serious | None | ⨁⨁⨁ Moderate |

**CI:** confidence interval

RCT: randomized controlled trial

#### Explanations

1. Might represent substantial heterogeneity, I^2^: 78%
2. The sample size of the included RCTs within the outcome allows the overall certainty to be moderate.
3. Might represent moderate heterogeneity, I^2^: 43%
4. Might represent substantial heterogeneity, I^2^: 75%
5. Sensitivity analysis reduced heterogeneity from the substantial level (I^2^: 64%) to that might be not important level (I^2^: 34%).
6. Sensitivity analysis reduced heterogeneity from the substantial level (I^2^: 76%) to that might be not important level (I^2^: 19%).
7. Sensitivity analysis reduced heterogeneity from the substantial level (I^2^: 73%) to that might be not important level (I^2^: 35%).
8. Wide CI: 1.26 [0.46,3.47]
9. Might represent considerable heterogeneity, I^2^: 96%
10. Might represent substantial heterogeneity, I^2^: 75%
11. Sensitivity analysis reduced heterogeneity from the substantial level (I^2^: 85%) to that might be not important level (I^2^: 0%).
12. Wide CI: 0.63 (0.14, 2.71)
13. Might represent considerable heterogeneity, I^2^: 93%
14. Might represent substantial heterogeneity, I^2^: 87%
15. Might represent substantial heterogeneity, I^2^: 67%
16. Wide CI: 2.12 (0.73, 6.12)
17. Sensitivity analysis reduced heterogeneity from the substantial level (I^2^: 75%) to that might be not important level (I^2^: 0%).
18. Sensitivity analysis resolved the issue of wide CI: 5.50 (2.98, 10.53), to be 7.56 (6.04, 9.47) instead.
19. Wide CI: 1.24 (0.45, 3.42)
20. Might represent considerable heterogeneity, I^2^: 95%
21. Wide CI: 3,30 (-0.60, 7.20)
22. Might represent considerable heterogeneity, I^2^: 92%
23. Wide CI: 3,41 (-0.88, 7.70)
24. Might represent considerable heterogeneity, I^2^: 99%
25. Wide CI: -1.39 (-3.13, 0.35)
26. Sensitivity analysis reduced heterogeneity from the substantial level (I^2^: 65%) to that might be not important level (I^2^: 0%).
27. Might represent considerable heterogeneity, I^2^: 99%
28. Wide CI: -1.38 (-4.23, 1.47)
29. Might represent substantial heterogeneity, I^2^: 81%
30. Wide CI: -1.00 (-3.79, 1.79)
31. Might represent considerable heterogeneity, I^2^: 97%
32. Might represent considerable heterogeneity, I^2^: 98%


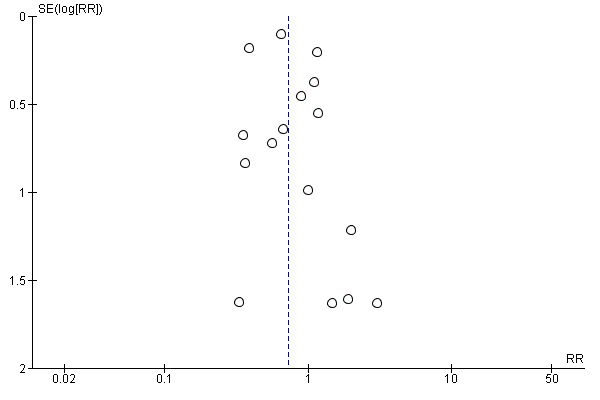


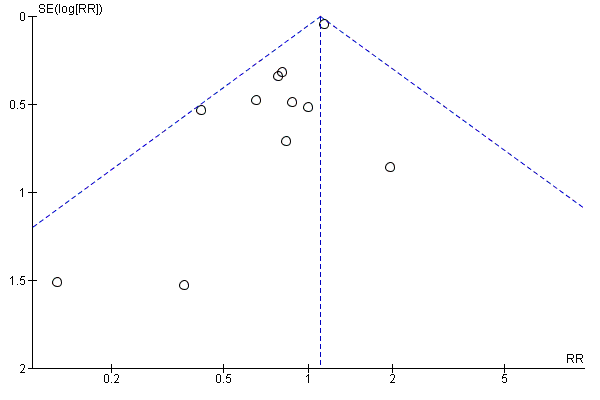
Supplementary fig. 1 funnel plot of studies that reported 30-day mortality

Supplementary fig. 2 funnel plot of studies that reported one-day mortality


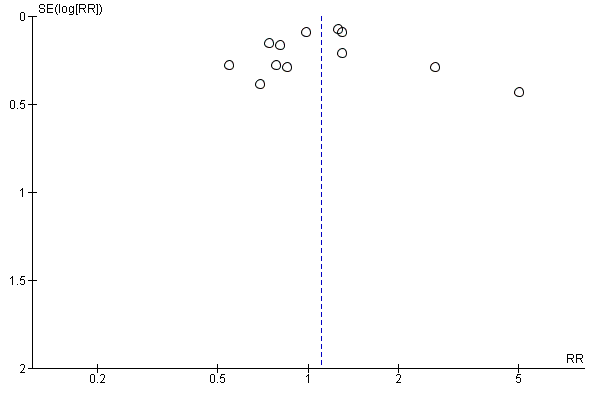
Supplementary fig. 3 funnel plot of studies that reported long-term mortality


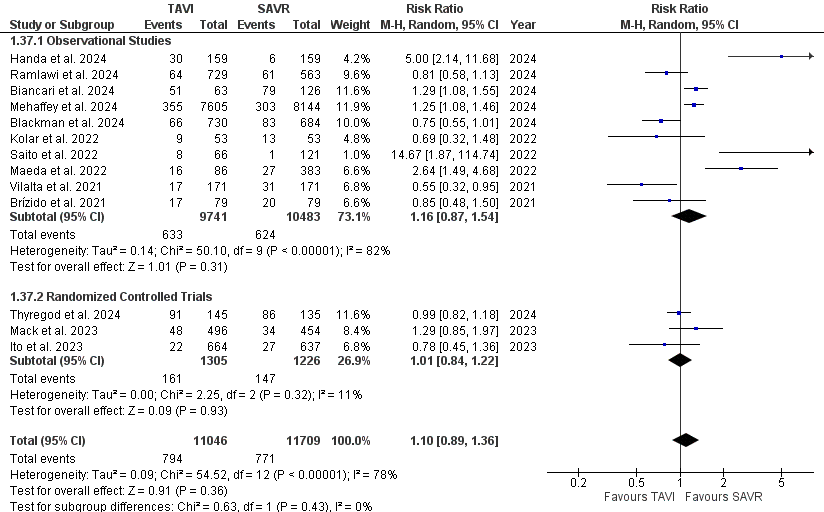
Supplementary fig. 4 forest plot of long-term mortality with a subgrouping based on study design


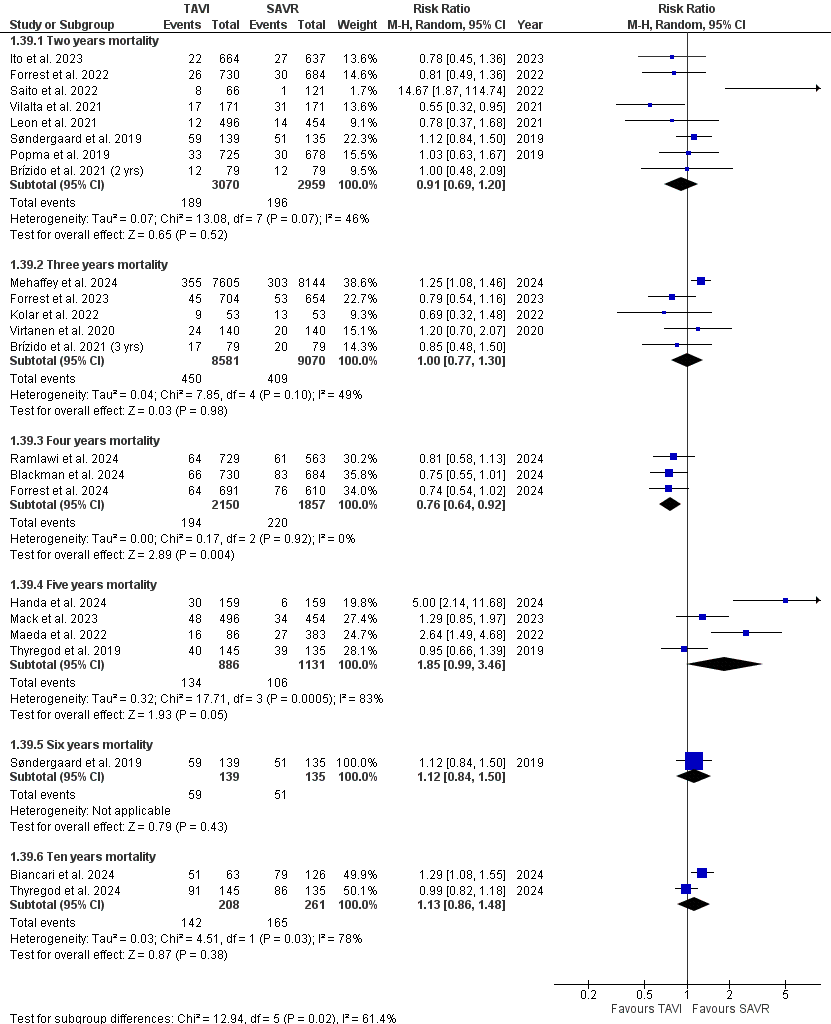


Supplementary fig. 5 forest plot of long-term mortality with a subgrouping based on the point of time were mortality rates measured


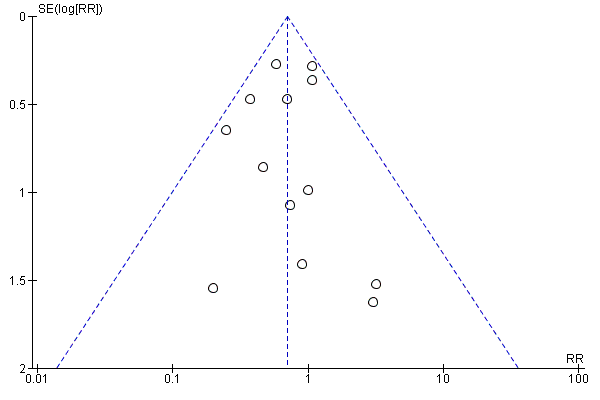
 Supplementary fig. 6 funnel plot of studies that reported 30-day stroke


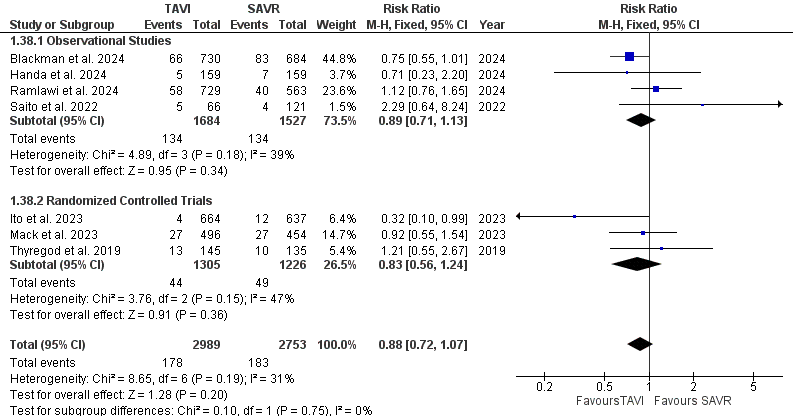


Supplementary fig. 7 forest plot of long-term stroke with a subgrouping based on study design


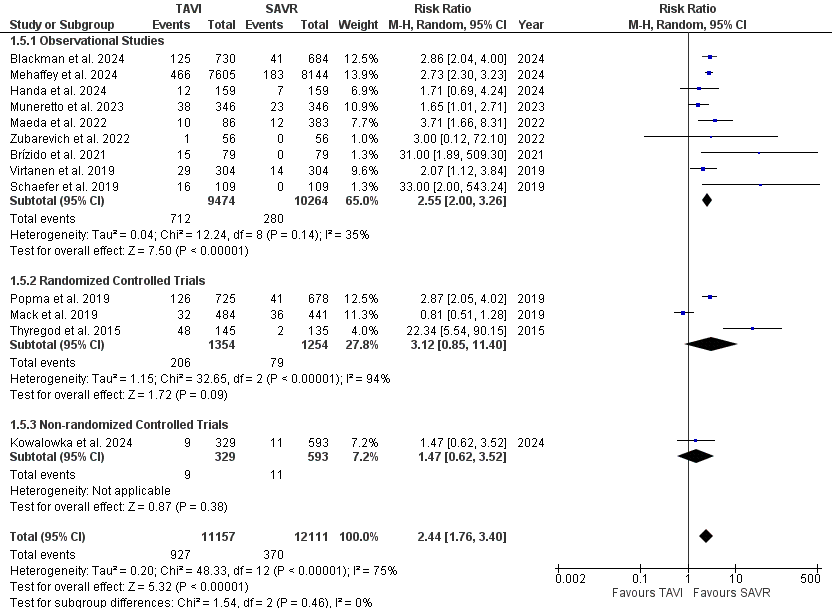
Supplementary fig. 8 forest plot of 30-days events of permanent pacemaker implantation (PPI) with a subgrouping based on study design

Supplementary fig. 9 forest plot of 30-days events of permanent pacemaker implantation (PPI) with a subgrouping based on the route used for the procedure
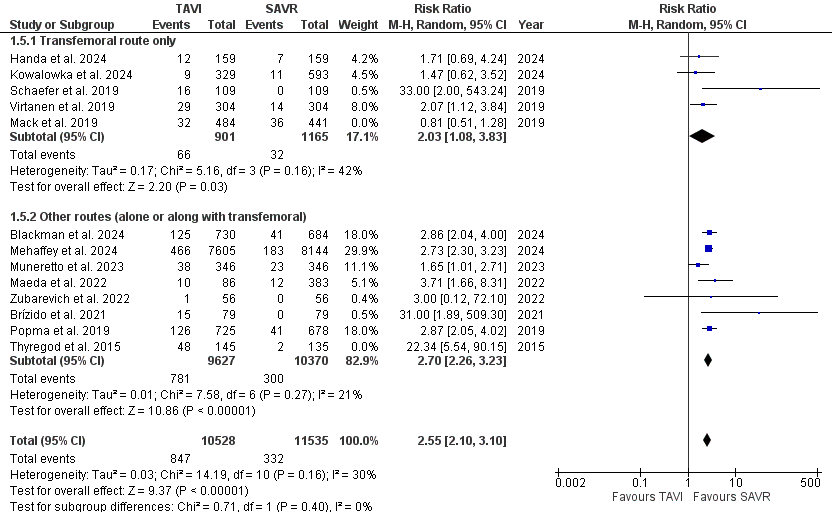
 and sensitivity analysis within each subgroup


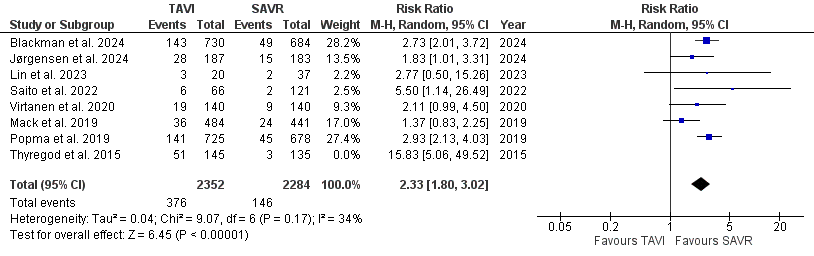


Supplementary fig. 10 sensitvity analysis of the meta-analysis model of one-year events of permanent pacemaker implantation (PPI)


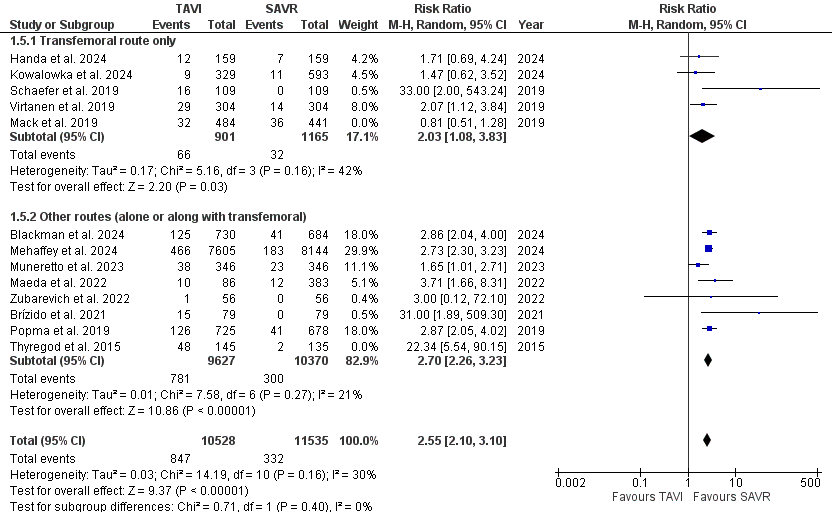


Supplementary fig. 11 forest plot of one-year events of permanent pacemaker implantation (PPI) with a subgrouping based on study design


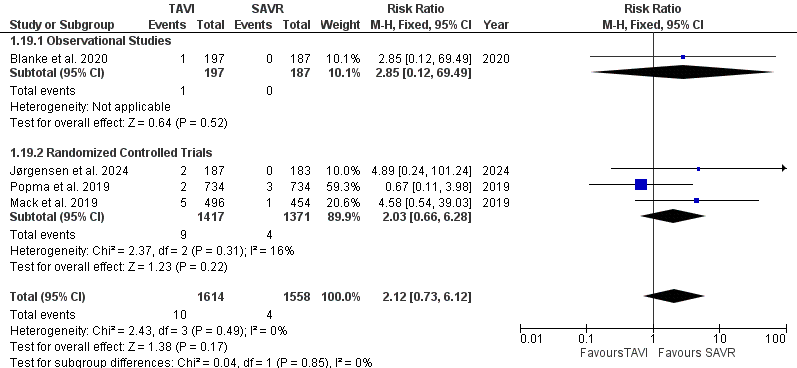
Supplementary fig. 12 forest plot of one-year events of valve thrombosis with a subgrouping based on study design


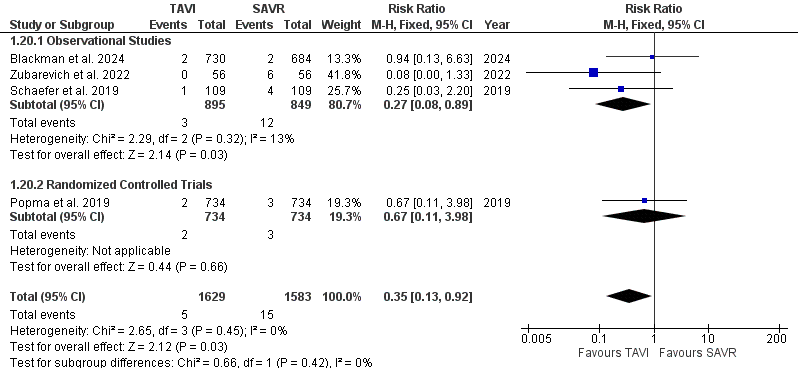
Supplementary fig. 13 forest plot of 30-days events of aortic reintervention with a subgrouping based on study design


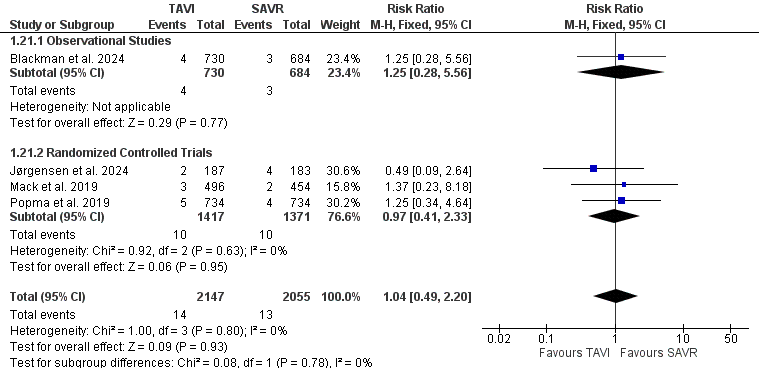
Supplementary fig. 14 forest plot of one-year events of aortic reintervention with a subgrouping based on study design


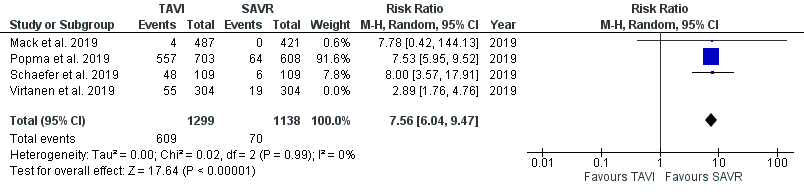
Supplementary fig. 15 sensitvity analysis of the meta-analysis model of 30-days events of paravalvular leakage (PVL)


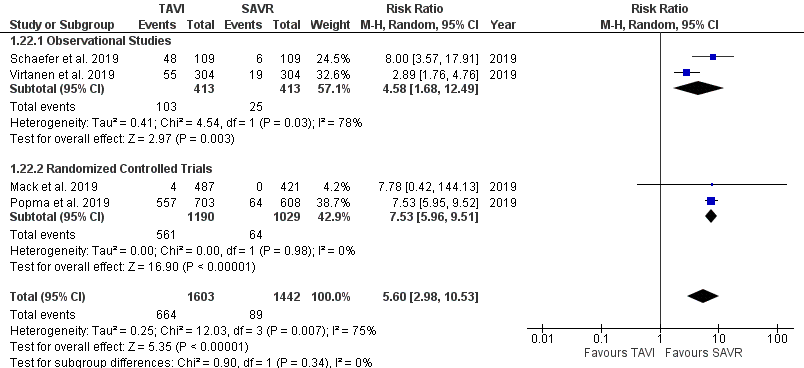


Supplementary fig. 16 forest plot of 30-days events of paravalvular leakage (PVL) with a subgrouping based on study design


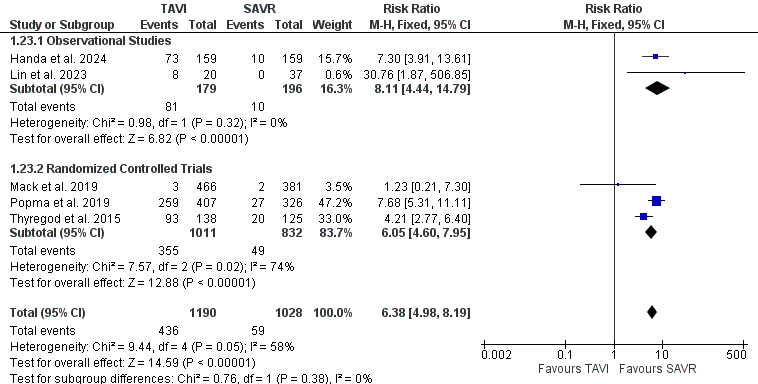


Supplementary fig. 17 forest plot of one-year events of paravalvular leakage (PVL) with a subgrouping based on study design


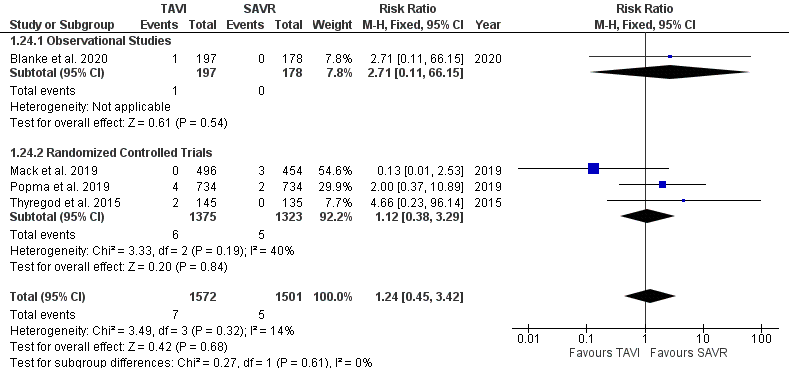


Supplementary fig. 18 forest plot of 30-days events of Transient Ischemic Attack (TIA) with a subgrouping based on study design


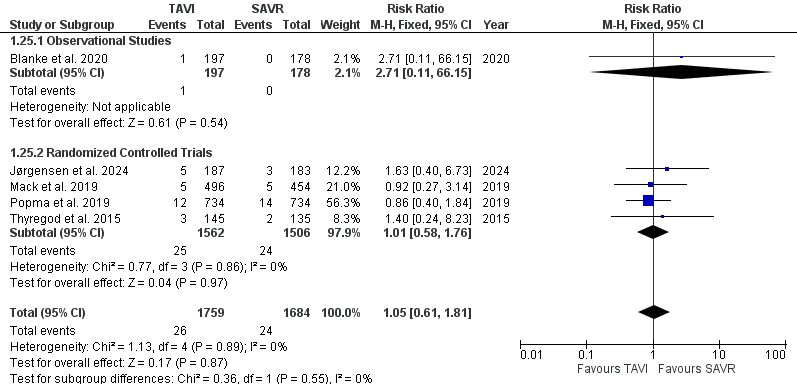


Supplementary fig. 19 forest plot of one-year events of Transient Ischemic Attack (TIA) with a subgrouping based on study design


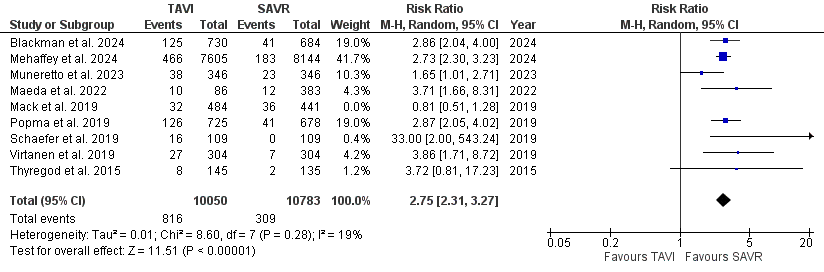


Supplementary fig. 20 sensitvity analysis of the meta-analysis model of 30-days events of major vascular complications


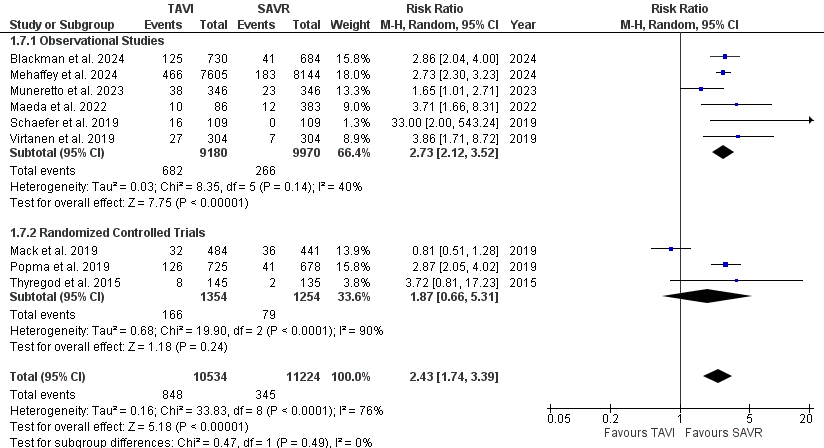


Supplementary fig. 21 forest plot of 30-days events of major vascular complications with a subgrouping based on study design


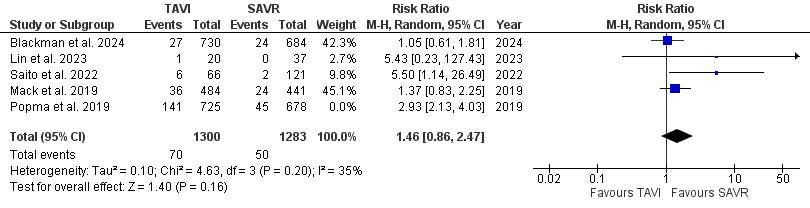


Supplementary fig. 22 sensitvity analysis of the meta-analysis model of one-year events of major vascular complications


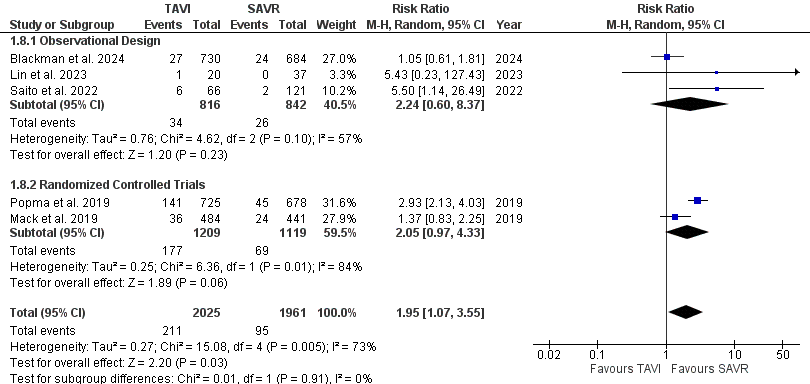


Supplementary fig. 23 forest plot of one-year events of major vascular complications with a subgrouping based on study design


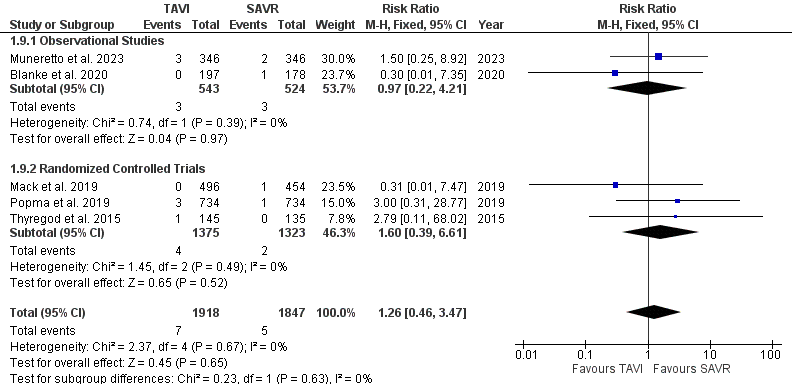


Supplementary fig. 24 forest plot of 30-days events of infective endocarditis with a subgrouping based on study design


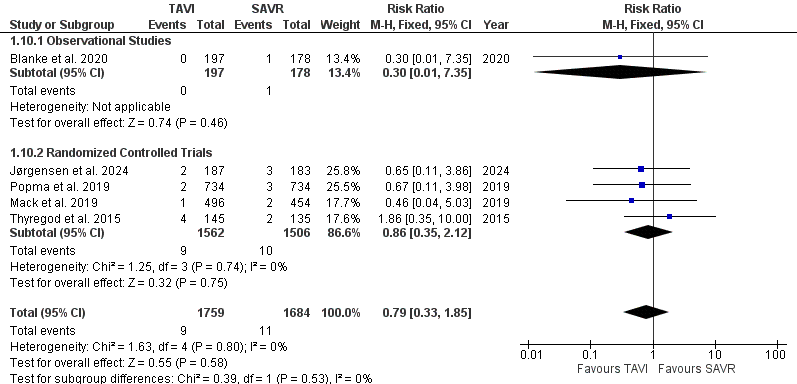


Supplementary fig. 25 forest plot of one-year events of infective endocarditis with a subgrouping based on study design


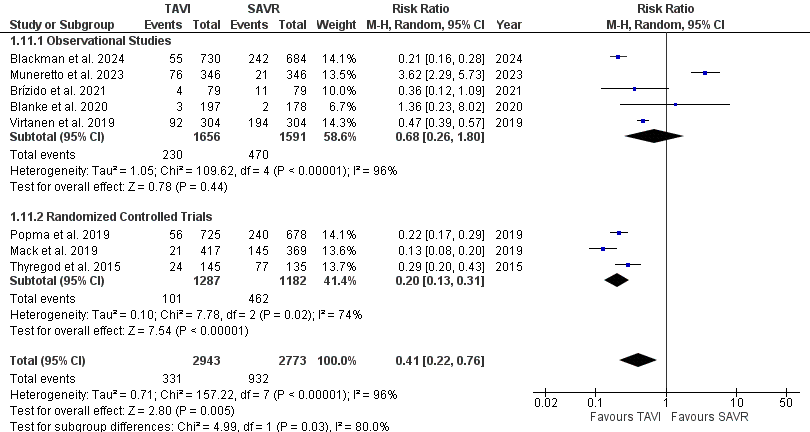


Supplementary fig. 26 forest plot of 30-days events of atrial fibrillation (AF) with a subgrouping based on study design


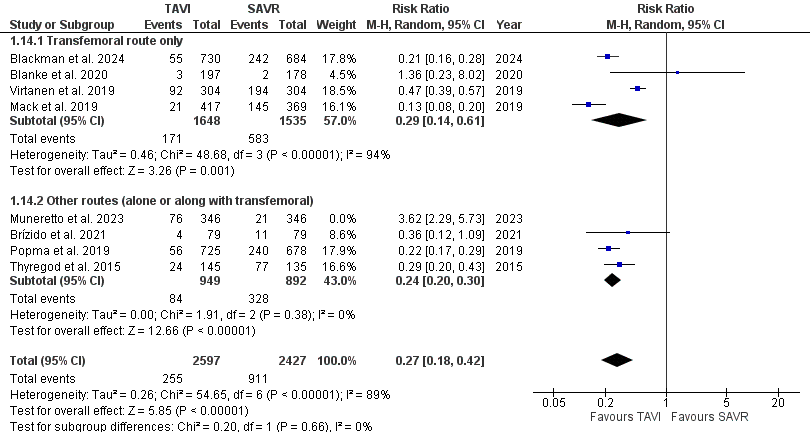


Supplementary fig. 27 forest plot of 30-days events of atrial fibrillation (AF) with a subgrouping based on the route used for the procedure and results of sensitivity analysis within the subgroup of other routes.


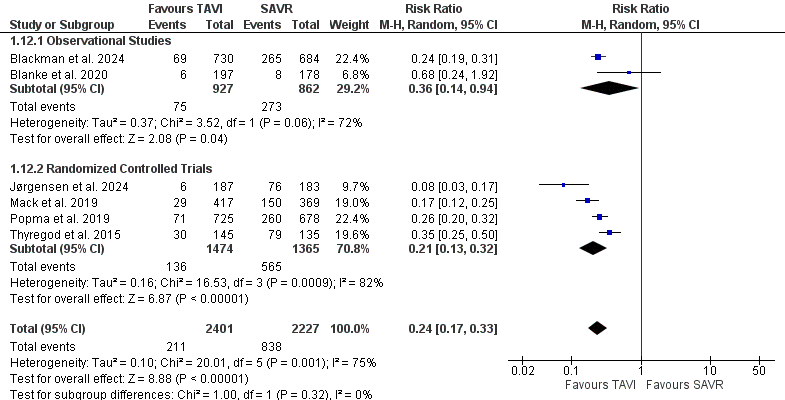


Supplementary fig. 28 forest plot of one-year events of atrial fibrillation (AF) with a subgrouping based on study design


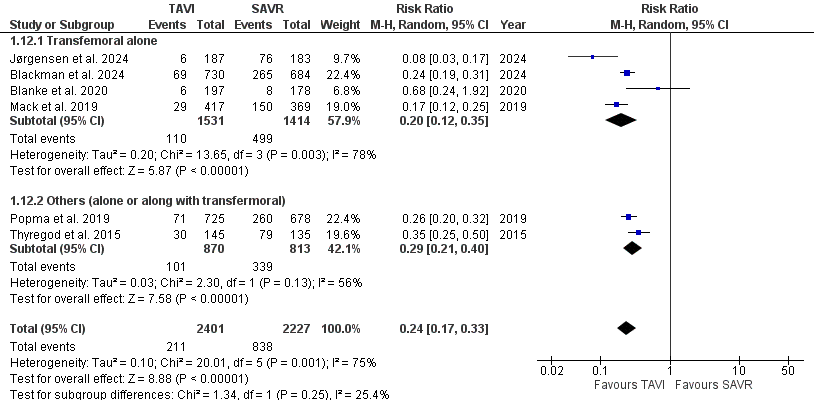


Supplementary fig. 29 forest plot of 30-days events of atrial fibrillation (AF) with a subgrouping based on the route used for the procedure


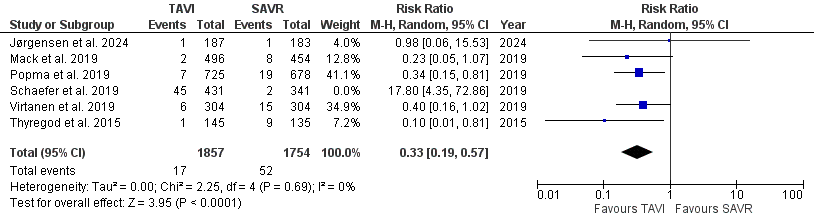


Supplementary fig. 30 sensitvity analysis of the meta-analysis model of one-year events of acute kidney injury (AKI)


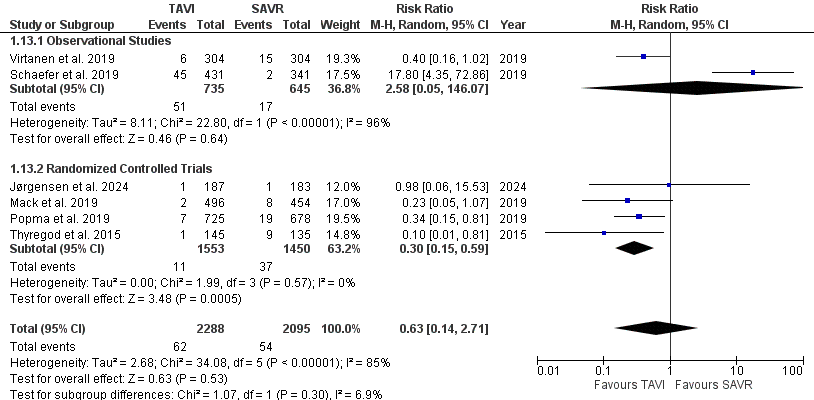


Supplementary fig. 31 forest plot of events of acute kidney injury (AKI) with a subgrouping based on study design


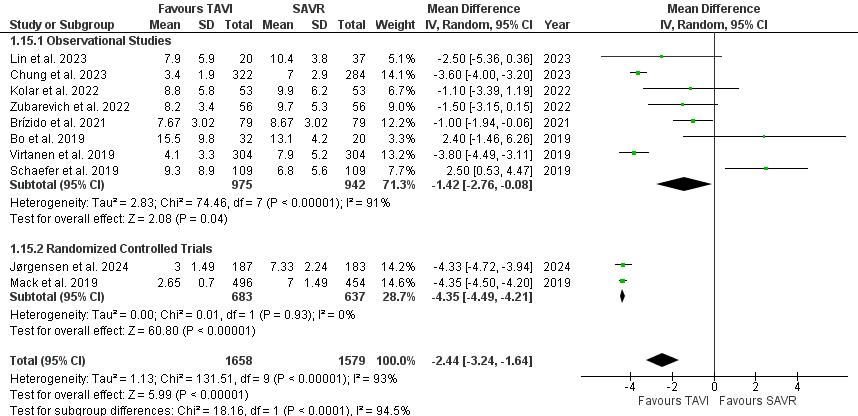


Supplementary fig. 32 forest plot of hospital stay with a subgrouping based on study design


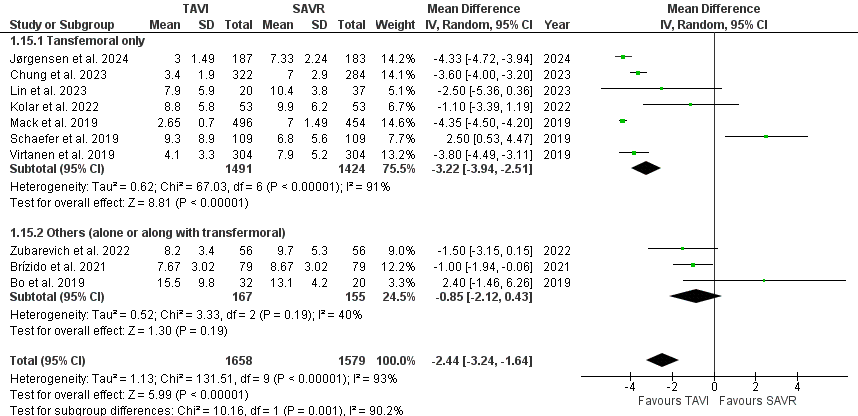


Supplementary fig. 33 forest plot of hospital stay with a subgrouping based on the route used for the procedure


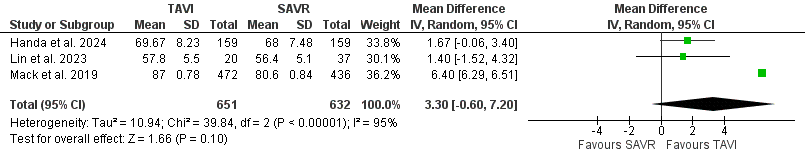


Supplementary fig. 34 forest plot of one-year ejection fraction with a subgrouping based on study design


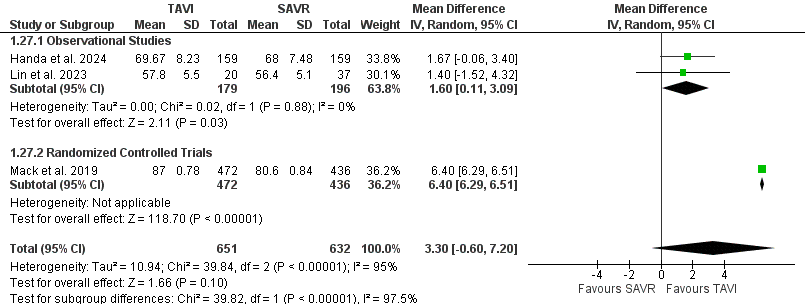


Supplementary fig. 35 forest plot of ejection fraction change from baseline after one year with a subgrouping based on study design


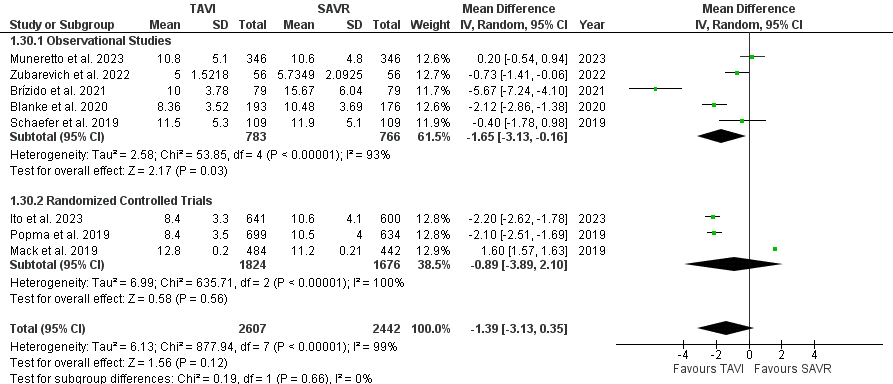


Supplementary fig. 36 forest plot of 30-days aortic pressure gradient with a subgrouping based on study design


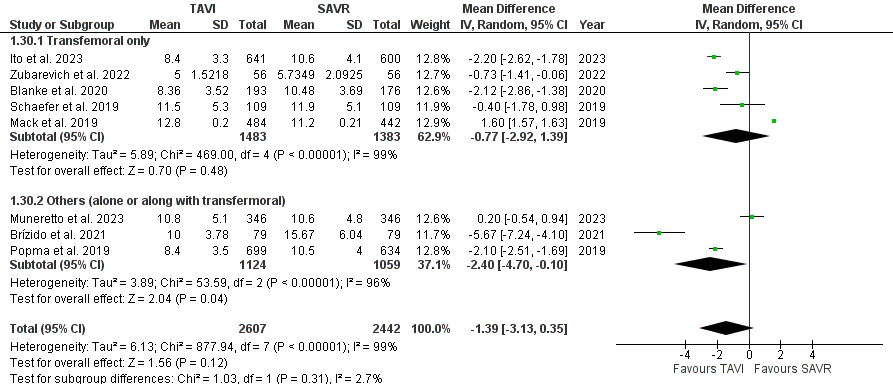


Supplementary fig. 37 forest plot of 30-days aortic pressure gradient with a subgrouping based on the route used for the procedure


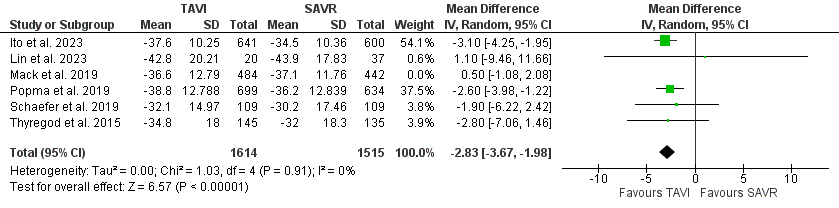


Supplementary fig. 38 sensitivity analysis of aortic pressure gradient change from baseline after 30 days


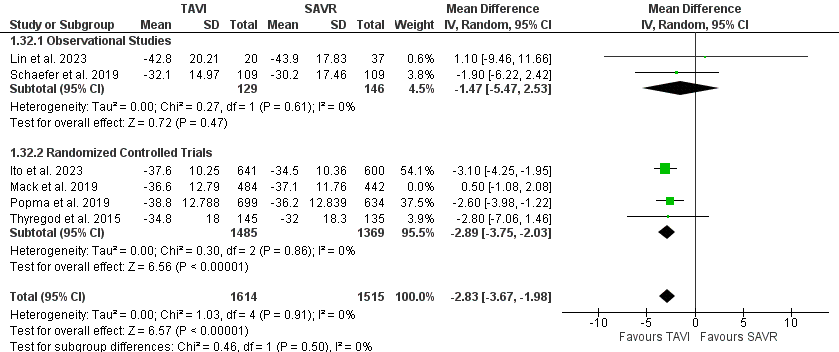


Supplementary fig. 39 forest plot of aortic pressure gradient change from baseline after 30 days with a subgrouping based on study design


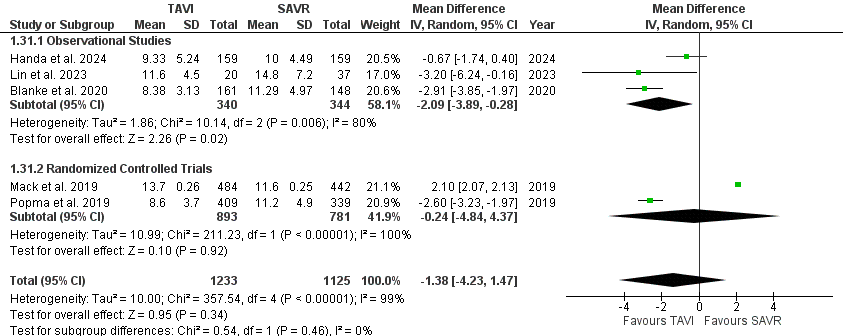


Supplementary fig. 40 forest plot of one-year aortic pressure gradient with a subgrouping based on study design


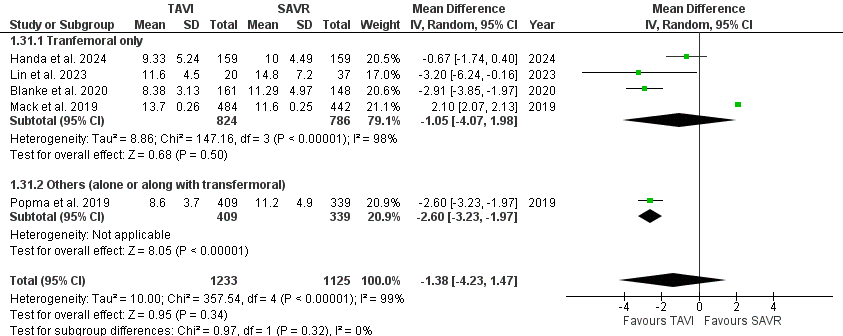


Supplementary fig. 41 forest plot of one-year aortic pressure gradient with a subgrouping based on the route used for the procedure


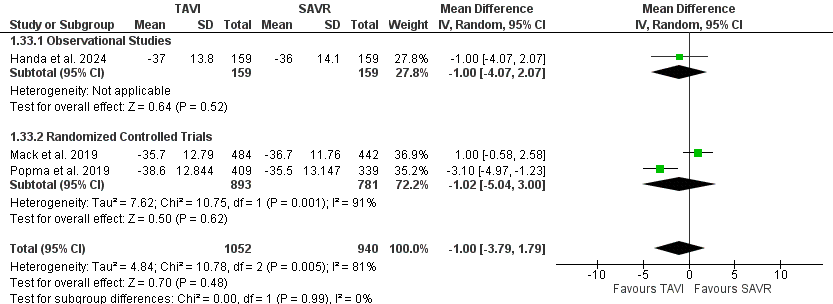


Supplementary fig. 42 forest plot of aortic pressure gradient change from baseline after one year with a subgrouping based on study design


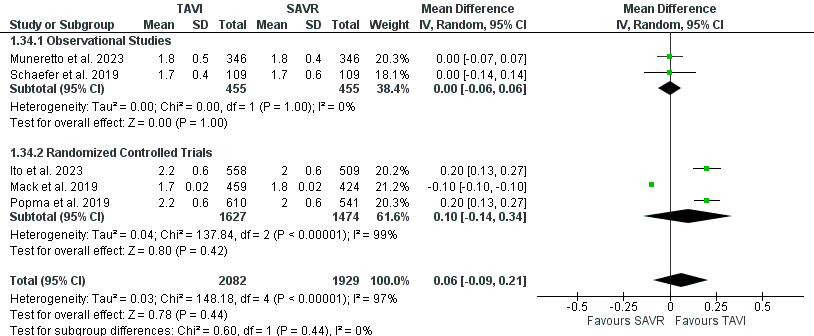


Supplementary fig. 43 forest plot of 30-days aortic valve area with a subgrouping based on study design


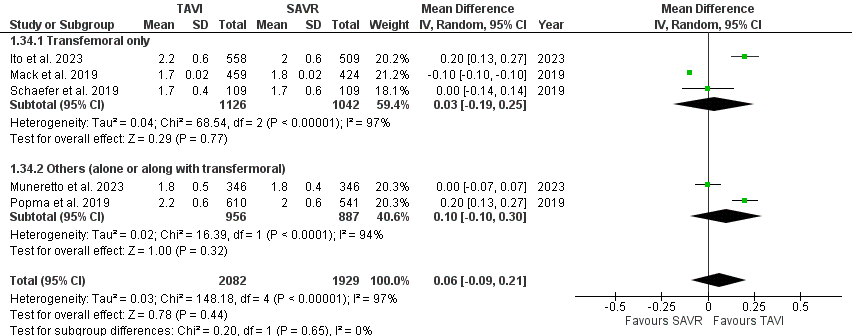


Supplementary fig. 44 forest plot of 30-days aortic valve area with a subgrouping based on the route used for the procedure


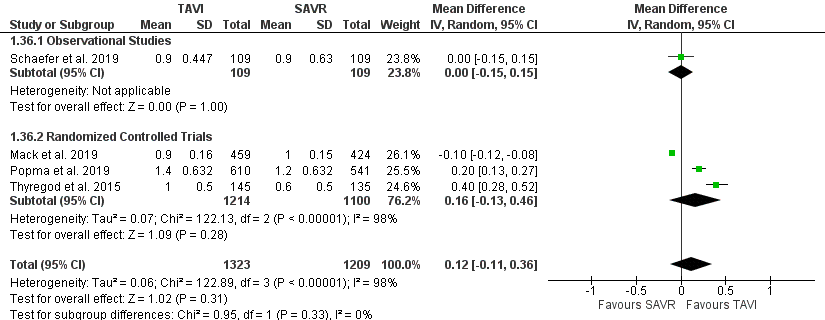


Supplementary fig. 45 forest plot of aortic valve area change from baseline after 30 days with a subgrouping based on study design


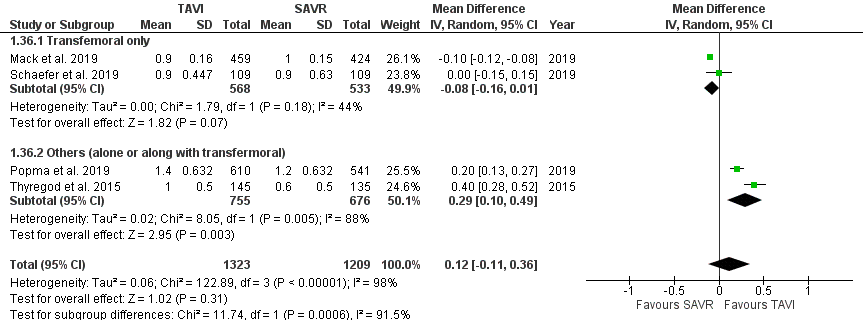


Supplementary fig. 46 forest plot of aortic valve area change from baseline after 30 days with a subgrouping based on the route used for the procedure
